# Sleep reveals dynamics integrating and segregating movement and stimulus representations in V1

**DOI:** 10.1038/s41467-026-75347-4

**Published:** 2026-07-21

**Authors:** Eliezyer Fermino de Oliveira, Soyoun Kim, Tian Season Qiu, Adrien Peyrache, Renata Batista-Brito, Lucas Sjulson

**Affiliations:** 1 Dominick P. Purpura Department of Neuroscience, Albert Einstein College of Medicine, Bronx, NY USA; 2 Montreal Neurological Institute, McGill University, Montreal, QC Canada; 3 Department of Psychiatry and Behavioral Sciences, Albert Einstein College of Medicine, Bronx, NY USA; 4 Department of Genetics, Albert Einstein College of Medicine, Bronx, NY USA; 5 Present Address: Nash Family Department of Neuroscience, Icahn School of Medicine at Mount Sinai, New York City, USA

**Keywords:** Sensorimotor processing, Computational neuroscience, Neuronal physiology

## Abstract

The primary visual cortex (V1) encodes multidimensional representations of ongoing movements, raising the question of how these signals are organized and interact with sensory representations within the same population. Here we addressed this question by using extracellular recordings in deep layers of mouse V1 to explore the relationship of movement and stimulus representations to intrinsic local circuit dynamics observed during non-REM (NREM) sleep. NREM revealed low-dimensional dynamics corresponding to an “on-manifold” subspace associated with the intrinsic manifold and an “off-manifold” subspace comprising dimensions suppressed by intrinsic dynamics, with remaining dimensions forming an “unstructured” subspace not systematically related to these dynamics. Movement and stimulus representations are both concentrated on-manifold, where they interact additively. However, stimulus representations are also concentrated off-manifold, where interference from movement-evoked activity is minimized. Off-manifold coding comprises population-sparse stimulus-evoked activity in chorister neurons, revealing an unexpected link between dimensionality, chorister/soloist cells, and sparse coding. Our findings suggest that intrinsic dynamics constrain neuronal activity in V1 and provide a structured substrate balancing the integration and segregation of movement and stimulus representations.

## Abstract (web summary)

How intrinsic dynamics structure sensory and movement representations in cortex remains unclear. Here, the authors show that these dynamics organize movement-stimulus interactions within low-dimensional manifolds while enabling sensory coding within separable off-manifold dimensions.

## Introduction

Recent studies using large-scale neuronal recordings have reported that activity in the primary visual cortex (V1) contains representations of movements and internal states^1–4^. This phenomenon is observed in both rodent and primate V1, though in primates the effect is weaker^5^ and mostly restricted to eye movements^6,7^. The function of these movement-related signals remains unclear, but the leading hypothesis is that they help correct stimulus representations for self-motion. This would require movement and stimulus signals to interact, but in a limited manner that minimizes interference with stimulus coding. How the brain achieves this balance remains unknown. Because all neural activity must evolve within the constraints imposed by local circuit dynamics, addressing this question requires understanding the intrinsic structure of population activity in V1.

Here, we investigated how movement and visual stimulus representations relate to low-dimensional intrinsic activity patterns in deep layers of mouse V1. We define “intrinsic” activity operationally as population patterns that occur in the absence of movements or visual stimuli, and are therefore putatively determined by local circuit dynamics within V1. To directly characterize these dynamics, we recorded during non-REM (NREM) sleep, an approach that has proven effective in studies of spatial navigation circuits^8–12^. NREM offers a cleaner window onto spontaneous cortical dynamics than wakefulness, which is rarely completely motionless, or REM sleep, which includes rapid eye movements that strongly modulate V1 activity^6,7^. Although replay events occur during NREM in some brain regions^13–16^, they represent only a small fraction of overall activity^17,18^ and do not dominate population structure, even in the hippocampus^19^, where replay is most well established. Thus, our use of NREM is not motivated by an interest in replay, but rather by its utility in revealing intrinsic activity patterns that reflect local circuit dynamics.

Analysis of mouse V1 activity during NREM sleep revealed low-dimensional dynamics that define three subspaces: an “on-manifold” subspace containing the intrinsic manifold, an “off-manifold” subspace comprising dimensions suppressed by intrinsic dynamics, and an “unstructured” subspace comprising the remaining dimensions. Importantly, intrinsic dynamics defined both dominant and suppressed directions of activity. Analyzing awake activity, we found that representations of movements and visual stimuli are both concentrated on-manifold, where they interact additively. However, stimulus information is also concentrated off-manifold, where interference from movement-related activity is minimized. Off-manifold coding emerges when “chorister” neurons, defined by strong coupling to population-dense activity during NREM, participate in sparse stimulus-evoked activity patterns. This coding scheme exploits the low-dimensional intrinsic dynamics of V1 to balance the integration and segregation of movement and stimulus representations.

## Results

### Leveraging NREM sleep to identify intrinsic manifold structure

**Fig. 1:**
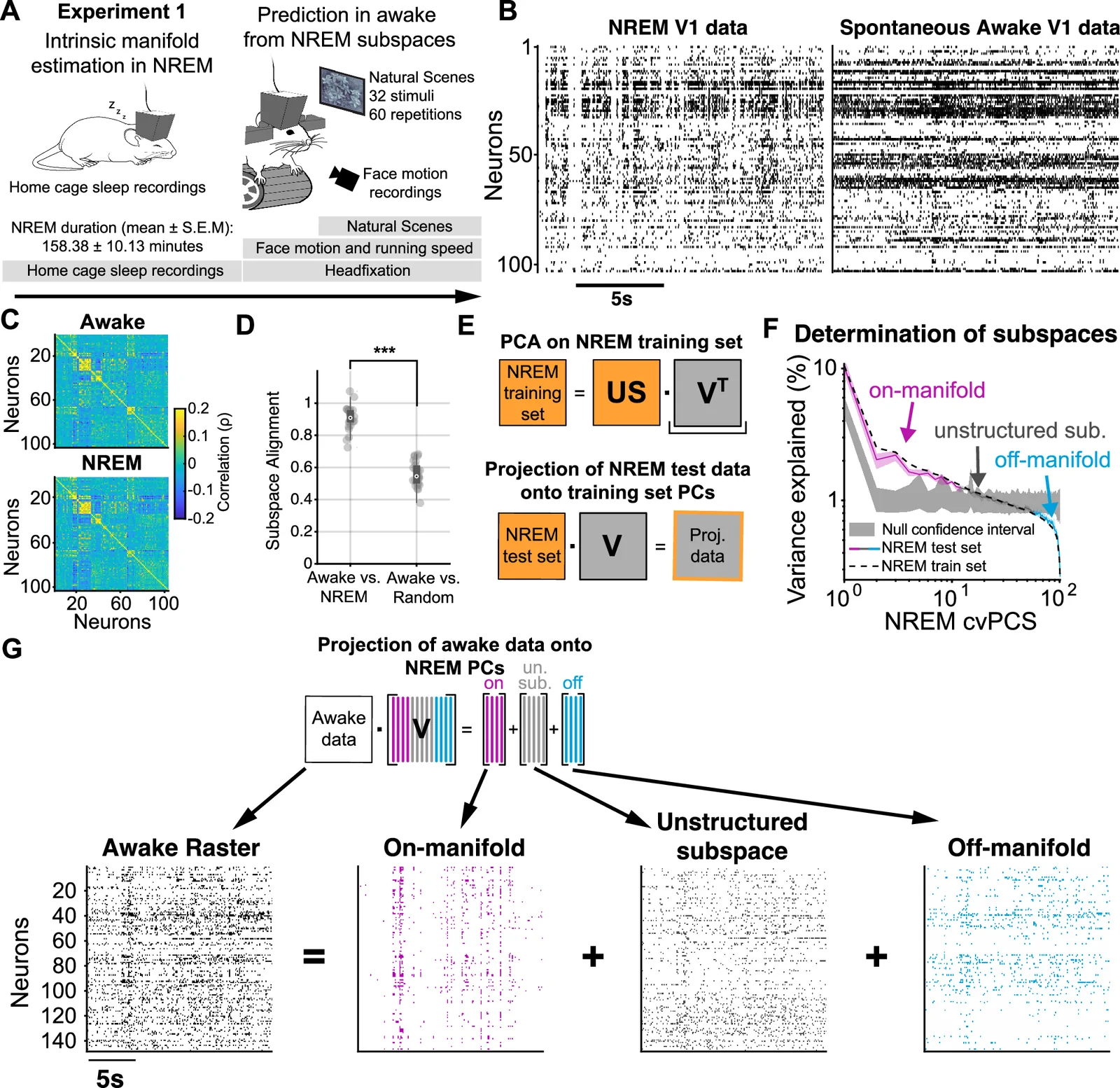
Using Non-REM (NREM) sleep to reveal latent intrinsic structure in awake V1 activity. **A** In Experiment 1, we recorded neuronal activity with chronic silicon probes in mouse V1 during home-cage sleep, followed by head-fixed recordings with natural visual scene stimuli and tracking of running speed and face motion. **B** Example recording showing similar V1 population activity in wake and NREM states. **C** Correlation matrices from awake and NREM states exhibit similar structure. **D** Spontaneous activity in NREM and wakefulness shows strong subspace alignment (see “Methods”, 0.91 ± 0.01, mean ± s.e.m., *n *= 26 sessions, *p *= 1.90  × 10^−18^, t(25) = 23.24, paired *t* test, two-sided), indicating that the intrinsic manifold identified during sleep remains prominent during wakefulness. Box plots show median, 25th-75th percentiles, and 1.5 × IQR whiskers. **E** Intrinsic manifolds were estimated using cross-validated PCA (cvPCA) by splitting NREM data into training and test sets and projecting the test set onto the training set PCs to estimate intrinsic population structure and distinguish structured variance from chance. **F** We operationally defined three subspaces: on-manifold (magenta), unstructured (gray), and off-manifold (cyan), which during NREM account for more, equal, or less variance than chance, respectively. The example shows a single session in mouse V1. Solid line and shaded area represent mean  ± s.e.m. **G** Representative data segment illustrating that V1 awake activity can be decomposed into components aligned with dominant, suppressed, and unstructured dimensions defined during NREM. Importantly, off-manifold activity is not simply activity outside the manifold, but is confined to specific low-dimensional directions defined by intrinsic dynamics. Panel A includes modified graphical elements adapted from SciDraw.io: “mouse sleeping” by Ethan Tyler and Lex Kravitz (10.5281/zenodo.3925977) and “Classical Conditioning Mouse” by Mackenzie Mathis (10.5281/zenodo.3925907), both licensed under Creative Commons Attribution 4.0 International (CC BY 4.0; https://creativecommons.org/licenses/by/4.0/). Changes were made to the original images.

To examine how movement-evoked, stimulus-evoked, and intrinsic activity relate to one another, we used NREM as a window into the intrinsic dynamics of V1. We chronically implanted 64- or 128-channel silicon probes into deep layers of V1 in C57BL/6J mice (see Methods). In each session of Experiment 1 (Fig. 11A), we recorded during sleep in the home cage for 158.38 ± 10.13 min, then head-fixed the mouse and continued recording while monitoring movements (running speed and facial motion) for 40 min. We then presented 32 natural visual scenes in random order 60 times each while continuing to monitor movements. These recordings (Fig. 1B) yielded an average of 160 units per session. We found that the population structure of V1 activity during NREM is mostly preserved during wakefulness (Fig. 1C, D - subspace alignment 0.62 ± 0.01, mean ± s.e.m., *n *= 26 recordings), consistent with previous results in other brain structures^8–10,12^, and this structure was not trivially explainable by differences in firing rates across cells (Supplementary Fig. S1). NREM enabled us to make more reliable estimates of the population structure in V1 than awake spontaneous activity (Supplementary Fig. S2A–C), and NREM activity patterns were lower-dimensional than during either wakefulness or REM (Supplementary Fig. S2D, E), suggesting that NREM provides the best estimate of the intrinsic activity manifold.

Our goal was to characterize how intrinsic dynamics structure the full neuronal state space, not only by defining the dominant low-dimensional manifold, but also by determining which dimensions are suppressed or unstructured. To do this, we used cross-validated PCA (cvPCA), which divides the NREM data into training and test sets and projects the test data onto the training-set principal components (Fig. 1E). To identify dimensions that are reliably expressed by intrinsic dynamics, we generated a null distribution by shuffling neuronal identities in the test set 10,000 times. Dimensions with variance exceeding this null were defined as “on-manifold” (Fig. 1F, magenta), corresponding to the dominant low-dimensional subspace that contains the intrinsic activity manifold. We then examined the remaining dimensions relative to this null distribution. Dimensions with variance below chance define an “off-manifold” subspace, reflecting patterns of activity that occur less often than expected and are therefore suppressed by intrinsic dynamics during NREM. In contrast, dimensions with variance near chance form an “unstructured” subspace, indicating activity that does not show a consistent relationship to the intrinsic dynamics (Fig. 11F). Using this framework, we organized the neuronal state space into on-manifold, off-manifold, and unstructured subspaces. We then projected awake activity into these subspaces (Fig. 1G, top), allowing us to decompose population activity into components aligned with dominant dynamics, suppressed directions, and unstructured activity (Fig. 1G, bottom). This decomposition enabled us to examine the encoding of movement and stimulus representations relative to the underlying intrinsic organization of V1 activity.

### Multidimensional movement representations are concentrated in the NREM-defined on-manifold subspace

**Fig. 2:**
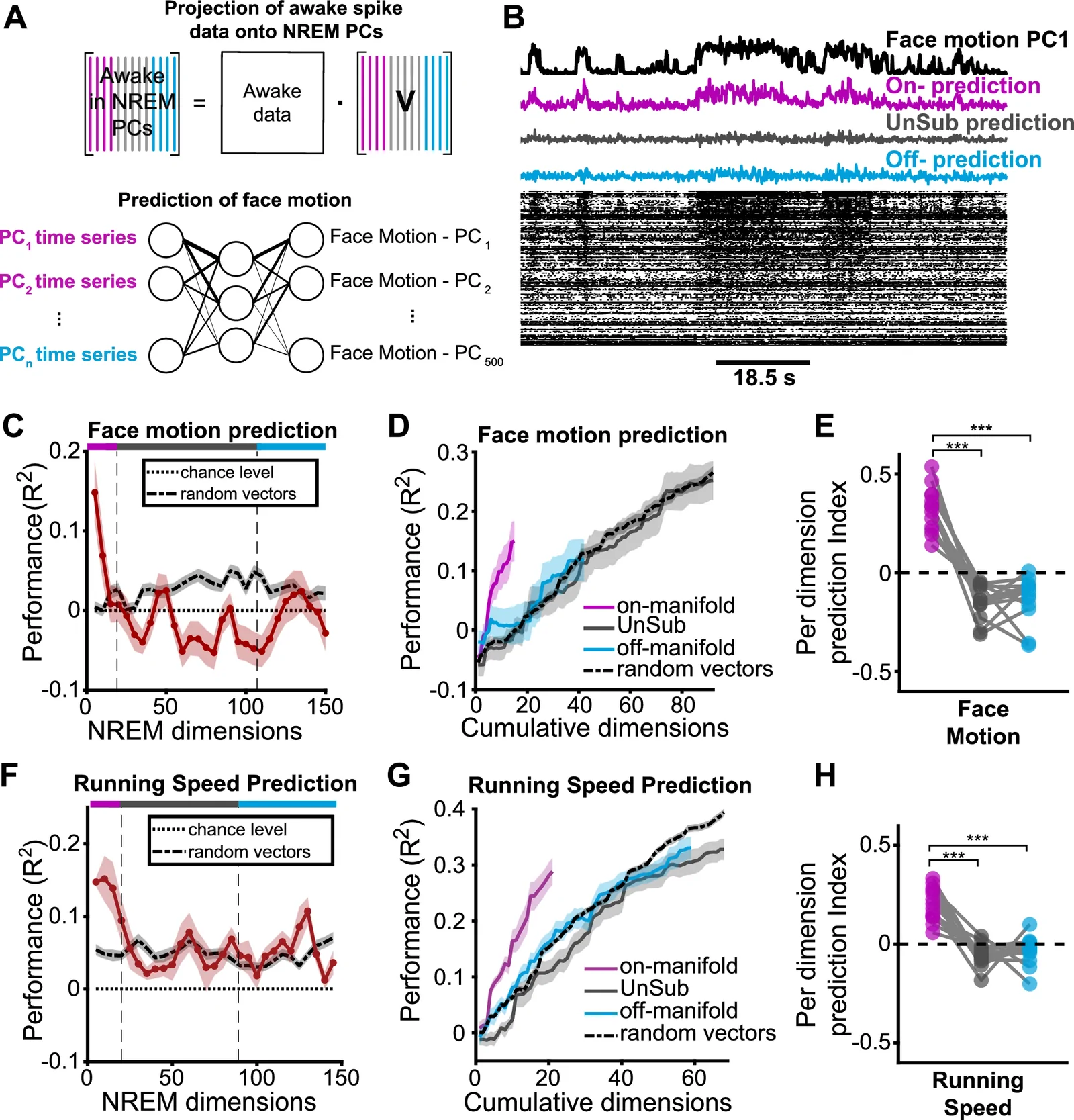
Movement representations are concentrated in the on-manifold subspace preserved during NREM. **A** We projected the neuronal data from head-fixed periods into the NREM subspaces and used reduced-rank regression to predict the face motion PCs. **B** Representative V1 raster during face motion, with neurons sorted by correlation with face motion PC1 (black). On-manifold activity (magenta) predicts face motion PC1 better than unstructured subspace (gray) or off-manifold (blue) subspaces of equal dimensionality. **C** Single-session prediction performance using sliding windows of 10 PCs; above-chance prediction occurs only in the on-manifold subspace. **D** Example of cumulative prediction performance with PCs in each subspace. Although the unstructured subspace predicts face motion best due to its high dimensionality, the on-manifold subspace shows substantially higher performance per dimension. **E** A per-dimension prediction index (relative to random subspaces) confirms that face motion representations are concentrated on-manifold (0.32 ± 0.03 vs. unstructured -0.14 ± 0.02 and off-manifold − 0.13 ± 0.03; *n *= 13 recording sessions, mean ± s.e.m.). Repeated Measures ANOVA F(2,24) = 62.64, *p *= 2.98 × 10^−10^ (**F**). Single-session example prediction performance of running speed using a sliding window, as shown in (**C**). Prediction quality is highest in the on-manifold subspace. **G** As in (**D**), the on-manifold dimensions show superior performance per dimension. **H** A per-dimension prediction index for running speed shows concentration in the on-manifold subspace. Prediction index: running speed on-manifold 0.20 ± 0.02, unstructured -0.05 ± 0.01, off-manifold − 0.03 ± 0.02, *n *= 14 recording sessions; mean ± s.e.m. Repeated Measures ANOVA F(2,26) = 42.47, *p *= 6.43 × 10^−9^ [All tests are two-sided, Tukey’s post hoc. ***p < 0.001]. Solid lines and shaded area represent mean  ± s.e.m., respectively.

First, we tested the relationship of movement-evoked activity to the three NREM-defined subspaces. We analyzed a video of the mouse’s face using FaceMap^20^ to extract 500 facial motion PCs that summarize facial movements. We then projected the awake neuronal activity into the three subspaces and used reduced rank regression to make cross-validated predictions of the facial motion PCs using the on-, unstructured subspace, and off-manifold activity (Fig. 2A). The best predictions of facial motion PCs were from on-manifold dimensions (Fig. 2B, C). Because the number of on-manifold dimensions is small, the total amount of facial motion information in the on-manifold space is less than in the higher-dimensional unstructured space (Fig. 2D). However, the amount of facial motion information per dimension is much higher in the on-manifold subspace (Fig. 2E, Prediction index: face motion: on-manifold 0.32 ± 0.03, unstructured subspace − 0.14 ± 0.02, off-manifold − 0.13 ± 0.03, *n *= 13; mean ± s.e.m.), suggesting concentration of the movement representation there. Repeating these analyses using running speed instead of facial motion yielded the same results (Fig. 2F–H Prediction index: running speed on-manifold 0.20 ± 0.02, unstructured subspace − 0.05 ± 0.01, off-manifold − 0.03 ± 0.02, *n *= 14; mean ± s.e.m.). The on-manifold movement representations were multidimensional (Supplementary Figs. S3A, B, S4A, B) and thus inconsistent with trivial explanations such as movement-correlated fluctuations in neuromodulator levels or overall firing rate (Supplementary Fig. S3C, D). Together, these data suggest that awake movements preferentially recruit the same intrinsic low-dimensional activity patterns that are present during NREM.

### Intrinsic local circuit dynamics constrain, but do not dictate, movement-evoked activity patterns

These findings raised two key questions. First, does the on-manifold structure of movement-evoked activity arise from the structure of the inputs themselves, or is it imposed by intrinsic local circuit dynamics? In other words, are movement-related inputs to V1 already aligned with the intrinsic manifold, or does the network compress a broader range of inputs into this low-dimensional subspace? Second, the accurate decoding of specific movements from V1 activity implies that their representations contain detailed kinematic information conveyed by long-range inputs from outside V1. How do these highly structured feedforward inputs interact with V1’s intrinsic local circuit dynamics?

**Fig. 3:**
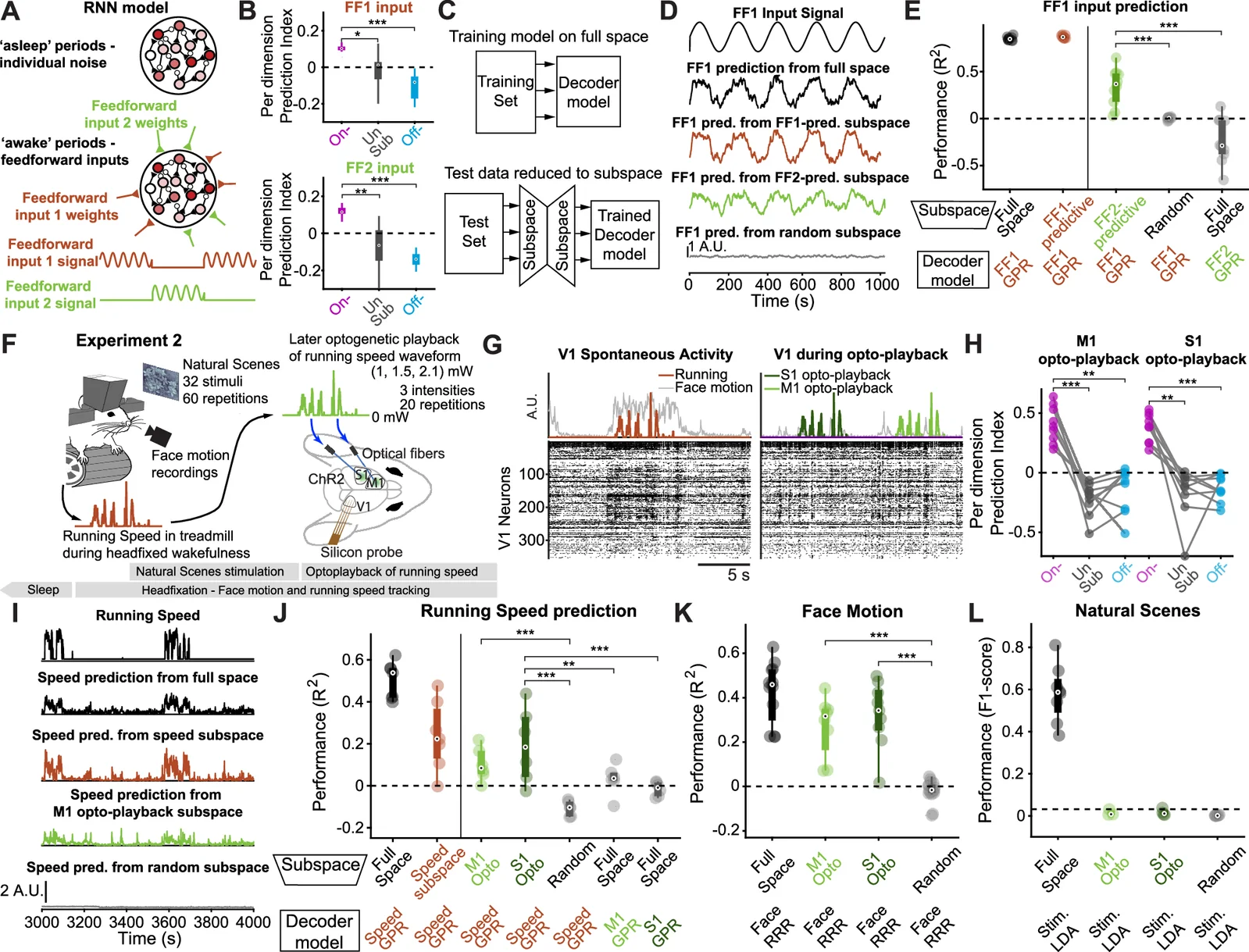
Intrinsic dynamics concentrate movement-evoked activity in overlapping subspaces but do not determine its structure. **A** Schematic of the RNN framework testing whether intrinsic recurrent dynamics constrain activity to a low-dimensional manifold independent of input structure. Intrinsic dynamics were measured without feedforward input, then responses to two random feedforward inputs (FF1, FF2) were examined. **B** RNN intrinsic local circuit interactions concentrate both inputs on-manifold (*n *= 10 repeats; RM ANOVA for FF1 input: F(2,18) = 12.78, *p *= 3.5 × 10^−4^; FF2 input: F(2,18) = 30.76, *p *= 1.56 × 10^−6^). **C** Cross-subspace decoding schematic. FF1 activity was projected into the optimal FF2 decoding subspace to test whether both inputs occupy overlapping subspaces. **D** Example showing that FF1 can be decoded from the subspace encoding FF2. **E** Cross-subspace decoding summary. FF1 could be predicted from the FF2 subspace above chance, indicating overlapping representational subspaces. However, decoders trained directly on FF2 generalized poorly to FF1, demonstrating distinct representations within those subspaces. (*n *= 10 repeats; Bonferroni corrected *t* tests: FF2 subspace vs. Random *p *= 7.8 × 10^−4^, FF2 subspace vs. Random *p *= 8.26 × 10^−6^). **F** In Experiment 2, we played back running speed traces as optogenetic experiments to contralateral M1 or S1 during V1 recordings. **G** Example V1 raster plots during natural running (*left*) and optoplayback of the recorded running speed (*right*). **H** M1 (*left*) and S1 (*right*) optoplayback representations are concentrated on-manifold (M1 and S1 *n *= 9 sessions. RM ANOVA for M1 optoplayback: F(2,16) = 29.29, *p *= 4.49 × 10^-6^; S1 optoplayback: F(2,16) = 17.71, *p *= 8.79 × 10^-5^). **I** Example illustrating that running speed and M1 optoplayback occupy overlapping subspaces. **J** Cross-subspace running-speed prediction summary. M1 and S1 optoplayback subspaces predicted running speed above chance, whereas decoders trained directly on optoplayback activity did not, indicating distinct representations within shared subspaces. (*n *= 6 sessions, Linear mixed-effects model; Random vs M1 subspace *p *= 3.34 × 10^-4^; M1 subspace vs S1 GPR *p *= 0.078; M1 GPR vs M1 subspace *p *= 0.52; Random vs S1 subspace *p *= 1.62 × 10^−6^; M1 GPR vs S1 subspace *p *= 6.51 × 10^−3^; S1 GPR vs S1 subspace *p *= 3.8 × 10^−4^). **K** Face motion representations also overlapped with optoplayback subspaces. Cross-subspace prediction from M1 and S1 optoplayback subspaces is better than random subspaces (M1 *n *= 8 sessions; S1 *n *= 9 sessions; linear mixed-effects model: Random vs M1 subspace *p *= 5.09 × 10^−7^; Random vs S1 subspace *p *= 1.24 × 10^−8^). **L** Stimulus representations did not overlap with the optoplayback subspace, suggesting distinct encoding. M1 and S1 combined, *n *= 7 sessions, *p *= 0.22, t(6) = 1.38, paired *t* test. All box plots show median, 25th-75th percentiles, and 1.5 × IQR whiskers. [All tests are two-sided, Tukey’s post hoc. *p < 0.05, **p < 0.01, ***p < 0.001] Panel (**F**) includes a modified graphical element adapted from SciDraw.io: “Mouse head schema” by Luigi Petrucco (10.5281/zenodo.3925903) and “Classical Conditioning Mouse” by Mackenzie Mathis (10.5281/zenodo.3925907), both licensed under Creative Commons Attribution 4.0 International (CC BY 4.0; https://creativecommons.org/licenses/by/4.0/).

To address these questions, we used a recurrent neural network (RNN) model (Fig. 3A, see “Methods”) to examine how intrinsic local circuit dynamics interact with two distinct feedforward inputs modeling different movements. This approach allows us to test whether intrinsic dynamics are sufficient, in principle, to impose low-dimensional structure on population activity independent of the detailed structure of the inputs. To drive activity in the network, each unit was injected with Gaussian random noise. To model different awake movements, we gave two distinct patterns of feedforward input, FF1 and FF2, which differed in their distribution of input weights across units. As with the experimental data (Figs. 1, 2), we used cvPCA to map the population structure of the RNN’s intrinsic activity in the absence of feedforward inputs, corresponding to NREM. If intrinsic dynamics impose the observed geometry of movement representations, two predictions follow: (1) activity driven by distinct inputs should be concentrated in the on-manifold subspace, and (2) representations of different inputs should occupy largely overlapping subspaces, reflecting a shared constraint imposed by the network. Consistent with these predictions, we found that both FF1 and FF2 were concentrated in the on-manifold subspace (Fig. 3B), similar to our experimental data (Fig. 2E, H). To test whether FF1 and FF2 were represented in overlapping subspaces, we used cross-subspace prediction (Fig. 3C), which entails attempting to decode FF1 from the subspace optimally encoding FF2. To accomplish this, we trained two partial least squares (PLS) decoders on RNN activity to predict FF1 and FF2, enabling us to find the RNN activity subspaces that optimally encode each signal. Similar to our experimental data (Supplementary Figs. S3A, B, S4A, B), the FF1- and FF2-encoding subspaces were multidimensional (dimensionality - FF1: 8.6 ± 0.22, FF2: 8.4 ± 0.3, mean ± s.e.m., *n *= 10 simulations) despite the fact that they were driven by one-dimensional signals. For cross-subspace prediction, we projected RNN activity from the FF1 test set into the FF2-predictive subspace before applying the FF1 decoder (Fig. 3C). As a negative control, we also used random subspaces of equal dimension to the FF2-predictive subspace. If FF1 and FF2 were encoded in distinct subspaces, the decoding performance would be equal for the FF2-predictive and random subspaces. However, we found that the FF2-predictive subspace was substantially better (Fig. 3D, E), indicating that FF1 and FF2 are encoded in largely overlapping subspaces. To address the role of the structured feedforward inputs in determining activity patterns, we tested whether the decoder trained on FF2 could successfully decode FF1. Despite the subspace overlap, the decoder failed to do so (Fig. 3E, right). Together, these results indicate that intrinsic local circuit dynamics are sufficient to constrain the population structure of activity by concentrating it on-manifold, while the detailed structure of the representation remains dependent on the specific pattern of feedforward inputs.

Although the RNN model suggests that V1 local circuit dynamics are sufficient, in principle, to reproduce our experimental results, it does not establish whether this is what occurs in vivo. We therefore asked whether the on-manifold structure persists when V1 is driven by inputs that lack the structured neural patterns associated with real movements. Accordingly, we performed optogenetic stimulation during chronic V1 recordings after injecting AAV5-CaMKII-ChR2 and implanting optical fibers into M1 and S1 contralateral to the recording probe (Experiment 2, Fig. 3F). If the on-manifold structure of movement-evoked activity is imposed by intrinsic dynamics, then even nonspecific inputs should be encoded on the intrinsic manifold. Our aim was to compare real movement-evoked activity with nonspecific optogenetically-evoked activity mimicking movements. We targeted contralateral M1 and S1 because they do not project directly to V1^21,22^, allowing us to avoid direct monosynaptic stimulation. Since the inputs carrying physiological movement signals to V1 are not fully known, our strategy was to mimic the distributed cortical activation that accompanies movement^1,2^, with the goal of indirectly activating the relevant long-range inputs. Thus, the optogenetic stimulation provides a test of whether the manifold is imposed by intrinsic dynamics rather than inherited from the structure of movement-related inputs. The logic of the experiment was as follows: if intrinsic dynamics constrain population activity, then NREM, real movement, and optogenetically-evoked activity should all share a similar on-manifold structure, even though the optogenetic inputs to V1 do not encode real movements. To generate these nonspecific input patterns, we used an “optoplayback” strategy: five-second segments of spontaneous running speed were recorded and played back as optogenetic stimuli to M1 or S1 while the animal was awake but not running (Fig. 3F). The optoplaybacks thus preserved the temporal structure of running but did not encode real movements because neurons were stimulated nonspecifically. Optoplaybacks alternated between M1 and S1, with 20 repetitions at each of three intensities (1, 1.5, and 2.1 mW; see Methods). This stimulation evoked multidimensional activity in V1 with a dimensionality comparable to real running (Supplementary Fig. S4A, B). The experimental results confirmed three key predictions of the RNN model (Fig. 3B, E). First, optoplayback-evoked activity was concentrated on-manifold (Fig. 3H). Second, real running speed could be decoded from either the M1 or S1 optoplayback-encoding subspaces (Fig. 3I, J). Third, decoders trained on optoplaybacks failed to decode real running speeds (Fig. 3J, right). Together, these results support the conclusion that intrinsic local circuit dynamics constrain the space of population activity, while the structure of external inputs determines the specific patterns within that space. Further supporting this hypothesis, the optoplayback subspaces also encoded facial motion (Fig. 3K). These effects were not attributable to optoplayback-induced movement, as optoplaybacks did not elicit detectable running or facial motion (Supplementary Fig. S4C), and the optoplayback waveforms were decodable only from neuronal activity, not movements (Supplementary Fig. S4D). Curiously, visual stimulus identity could not be decoded from the optoplayback subspaces (Fig. 3L), raising the question of how the encoding of movements differs from visual stimuli.

### Natural visual scene representations are concentrated in both the on- and off-manifold subspaces

**Fig. 4:**
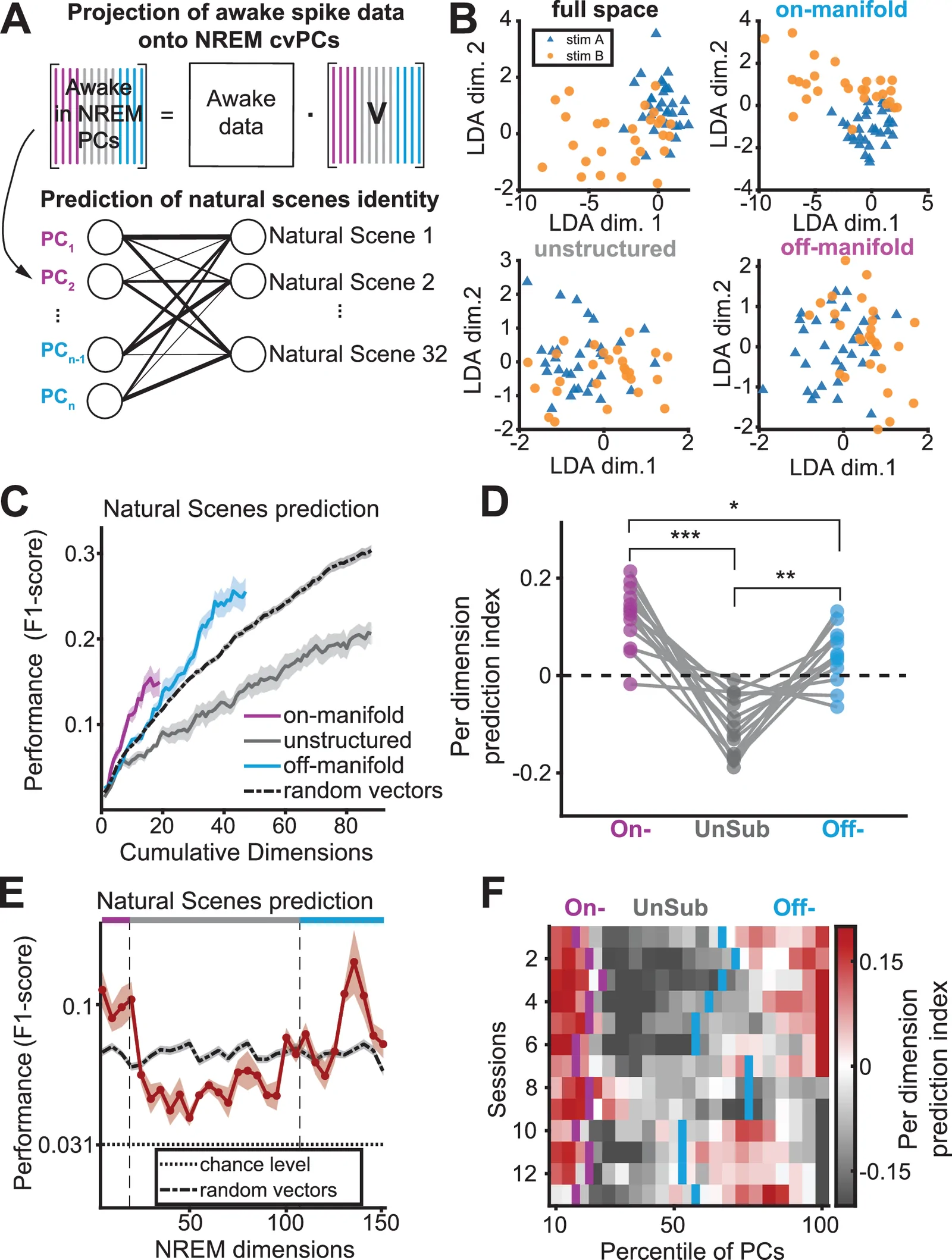
Visual stimulus information is concentrated in both on-manifold and off-manifold subspaces. **A** We projected neuronal data from awake, head-fixed periods into the NREM-defined subspaces and then used a linear discriminant analysis (LDA) model to predict the identities of the 32 different natural scene stimuli. **B** Representative cross-validated separation of two random natural scene presentations (stimulus A and B) in the top two LDA dimensions of either the full space or the on-/off-manifold and unstructured subspaces. The number of dimensions in the unstructured and off-manifold subspaces are matched for comparison. Note that stimulus separation is worse in the unstructured subspace. **C** Single session example of cumulative prediction performance with PCs in each subspace. Note that the on-manifold subspace has the most stimulus information per dimension, but the off-manifold subspace has the most stimulus information overall. Solid lines and shaded areas represent mean ± s.e.m., respectively. **D** Across-session summary showing that on- and off-manifold subspaces contained more stimulus information per dimension than the unstructured subspace. Prediction index: on-manifold 0.12 ± 0.02, unstructured − 0.10 ± 0.02, off-manifold 0.04 ± 0.02, *n *= 13 recording sessions; mean ± s.e.m. Repeated Measures ANOVA F(2,24) = 31.23, *p *= 2.09 × 10^−7^. **E** Single session example prediction performance of natural scenes using a sliding window of 10 NREM PCs. Prediction performance better than a random subspace was found only in PCs from the on- and off-manifold subspaces. Solid lines and shaded areas represent mean ± s.e.m., respectively. **F** Our operational definitions of the on-, off-manifold, and unstructured subspaces correspond approximately to the boundaries of higher and lower prediction indices for natural scenes. Magenta and cyan lines denote the limits of the on- and off-manifold subspaces, respectively. [All tests are two-sided, Tukey’s post hoc. *p < 0.05, **p < 0.01, ***p < 0.001.].

To address this next question, we analyzed the data collected in Experiment 1 to test which NREM-defined subspaces encode visual scene identity in the awake state (Fig. 4A). After projecting the awake data into the three subspaces, we trained an unnormalized linear discriminant (LDA, see “Methods”) decoder on held-out training data to predict the stimulus identity. Unexpectedly, we found that stimulus identity was preferentially encoded in both the on- and off-manifold subspaces (Fig. 4B–D). This was not attributable to nonspecific phenomena such as global firing rate modulation (Supplementary Fig. S5A), up and down state transitions (Supplementary Fig. S5B), putative fast-spiking interneurons (Supplementary Fig. S5C), or differences in firing rate between distinct neuronal subpopulations (Supplementary Fig. S5D). Combining the on- and off-manifold subspaces improved prediction performance further (Supplementary Fig. S6A, B) without reducing the predictive performance per dimension (Supplementary Fig. S6C), suggesting the two subspaces encode non-redundant information. The concentration of information per dimension is highest in on-manifold dimensions (Fig. 4D), but because the off-manifold subspace is higher dimensional, substantially more stimulus information is encoded there (Fig. 4C). To verify this was not an artifact of our heuristic definitions of the on/off/unstructured subspace boundaries, we assessed the decoding performance of the cvPCA dimensions in sliding bins of 10 cvPCs (see “Methods”). Decoding performance was highest for both high- and low-variance dimensions and lowest for intermediate ones, aligning approximately with the on-/off-manifold and unstructured subspace boundaries (Fig. 4E, F). We also addressed the possibility that off-manifold stimulus coding might be trivially explainable by activity in visually-driven neurons that are silent during NREM and therefore contribute little variance to cvPCA. Our findings were inconsistent with this. First, neurons with high off-manifold participation had higher firing rates during NREM than unstructured subspace neurons (see “Methods”, Supplementary Fig. S7A). Second, firing rate increases during wakefulness were similar across neurons regardless of subspace participation (Supplementary Fig. S7B). Finally, cells encoding the most stimulus information exhibited higher firing rates during NREM (Supplementary Fig. S7C, D).

### The on- and off-manifold subspaces correspond to dense and sparse activity in chorister neurons

**Fig. 5:**
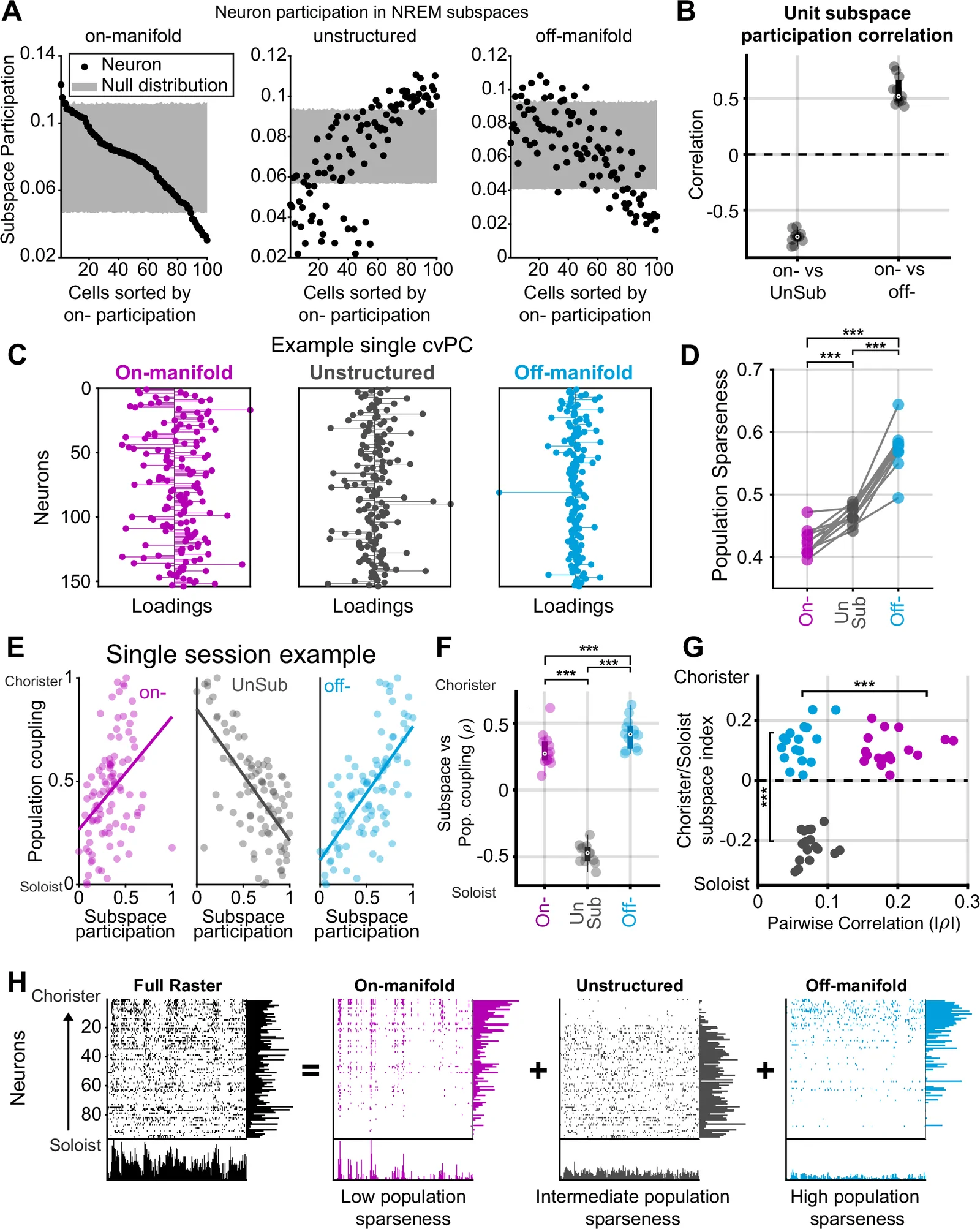
On- and off-manifold activity arise when chorister neurons participate in population-dense and population-sparse activity patterns. **A** Single-session example showing that many neurons (black dots) participate more in specific subspaces than expected by chance (gray is null). On-manifold participation is negatively correlated with unstructured subspace and positively with off-manifold participation. **B** Across sessions (*n *= 11), on-manifold participation correlates negatively with unstructured subspace and positively with off-manifold participation. **C** cvPCA loadings reveal lower population sparseness (i.e., more cells participate) in on-manifold dimensions and higher in off-manifold dimensions. **D** This pattern holds across sessions (*n *= 11 recording sessions; RM ANOVA F(2,20) = 198.56, *p *= 6.42 × 10^−14^). **E** Single-session example showing that on- and off-manifold subspaces are dominated by “chorister” neurons defined by strong population coupling during NREM. Conversely, the unstructured subspace is dominated by “soloists” defined by low NREM population coupling (each marker represents one neuron). **F** This pattern holds across sessions (*n *= 13 recording sessions; RM ANOVA F(2,24) = 264.51, *p *= 4.46 × 10^−17^). **G** Off-manifold activity reflects choristers participating in population-sparse activity (*n *= 17 sessions. Pairwise correlation: RM ANOVA F(2,32) = 169.68, *p *= 9.24 × 10^−18^; Chorister/Soloist Index: RM ANOVA F(2,32) = 248.05, *p *= 3.30 × 10^−20^). Each color-coded marker represents a subspace in one recording session. High pairwise correlation indicates low population sparseness. **H** Representative example illustrating that on-manifold activity consists of choristers firing with low population sparseness, unstructured subspace is soloists with low sparseness, and off-manifold is choristers with high sparseness. All box plots show median, 25th-75th percentiles, and 1.5 × IQR whiskers. [All tests are two sided, Tukey’s post hoc. ***p < 0.001].

We next investigated the nature of neuronal activity patterns occupying the three NREM-defined subspaces. We found that every neuron participates in all three subspaces, but individual neurons are biased to participate in certain subspaces more than expected by chance (*i.e*., if PC loadings were shuffled, Fig. 5A). Moreover, the same neurons with strong on-manifold participation also exhibited stronger off-manifold participation and weaker unstructured subspace participation (Fig. 5B), suggesting there may be distinct neuronal subpopulations that preferentially participate in the different subspaces. Our next clue to the nature of the three subspaces was the observation that off-manifold PCs had the highest population sparseness, e.g., the fewest cells participated in each PC (Fig. 5C, D, population sparseness: on-manifold 0.42 ± 0.01, unstructured subspace 0.47 ± 0.004, off-manifold 0.57 ± 0.01, *n *= 11 sessions). We suspected this could be connected to previous work^23^ reporting that cortical neurons exhibit a wide range of population coupling strengths, lying on a continuum from “choristers,” whose spontaneous activity is tightly coupled to the population, to “soloists,” whose spontaneous activity is largely independent. Where on the chorister-soloist axis an individual neuron lies is not merely a description of its activity at a given moment in time, but rather a stable long-term property associated with differences in anatomical connectivity^24^. We thus identified choristers and soloists by using population coupling during NREM (see “Methods”), then tested the relationship of choristers and soloists to the three subspaces. We found that the on- and off-manifold subspaces contained mostly activity in choristers, and the unstructured subspace contained mostly activity in soloists (Fig. 5E, F). Together, these results indicate that on-manifold activity is dominated by chorister neurons engaged in population-dense activity patterns, whereas unstructured subspace activity arises from soloist neurons engaged in population-sparse patterns. Unexpectedly, off-manifold activity reflects choristers participating in population-sparse patterns (Fig. 5G), i.e., when they fire decoupled from the population. These distinctions are apparent in Fig. 5H, which shows a raster plot of awake data projected into the three subspaces, with rows sorted by chorister/soloist index. Because choristers are strongly coupled to spontaneous population activity, they rarely participate in sparse patterns in the absence of visual stimuli, explaining why off-manifold activity is observed at below-chance levels during NREM. However, stimulus-evoked activity in V1 is population-sparse^25^, even in choristers, allowing visual stimuli to be encoded off-manifold (Fig. 4).

### On-manifold interactions between movement and stimulus representations are mediated by multiplexing in chorister neurons

**Fig. 6:**
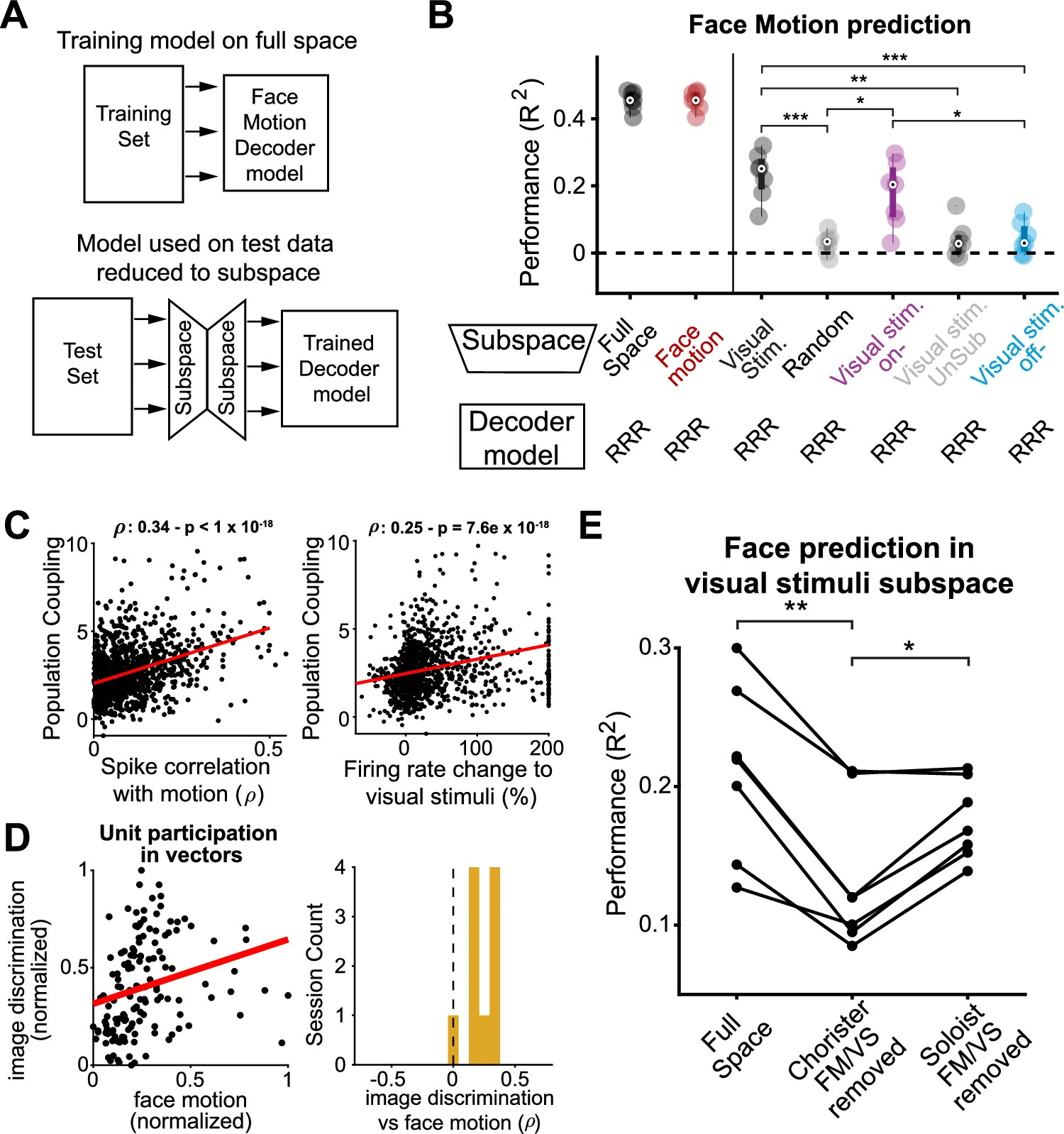
Movement and stimulus representations exhibit additive on-manifold interactions mediated by multiplexing in chorister neurons. **A** To test whether movement representations extend into the stimulus-encoding subspace, we performed cross-subspace prediction using a face motion decoder on neuronal activity projected into the subspace optimally encoding stimulus identity. **B** Face motion was predicted far better from the visual stimulation subspace than from a random subspace, with the interaction localized to the on-manifold subspace (*n *= 7 recording sessions; RM ANOVA F(4,24) = 30.05, *p *= 4.96 × 10^−9^). Box plots show median, 25th-75th percentiles, and 1.5 × IQR whiskers. **C** Neurons with stronger population coupling (choristers) showed stronger modulation by both movements (*p* < 1 × 10^−18^, *ρ *= 0.34, *n *= 1187 units) and visual stimuli (*p *= 7.6 × 10^−18^, *ρ *= 0.25, *n *= 1180 units). Spearman correlation statistical test, two-sided. **D** Neurons encoding face motion were also more likely to encode stimulus identity (single session *ρ *= 0.25, *p *= 2.1 × 10^−3^). (*Right*) Across session summary (n = 10 recording sessions, *t* test *p* = 4.32  × 10^−4^, correlation 0.21 ± 0.04). **E** Regressing out face-motion and stimulus modulation from choristers (top 30% of coupling) impaired face-motion prediction (*R*^2 ^= 0.13 ± 0.02, *R*^2 ^= 0.21 ± 0.02 for original data), whereas removing it from soloists (bottom 30%) had no effect (*R*^2 ^= 0.17 ± 0.01; *n *= 7 recording sessions; RM ANOVA F(2,12) = 20.48, *p *= 1.35 × 10^−4^). This suggests that multiplexing in choristers mediates movement-stimulus interactions. [All tests are two-sided, Tukey’s post hoc. Mean ± s.e.m. *p < 0.05, **p < 0.01, ***p < 0.001.].

Movement representations in V1 presumably exist because they contribute to visual processing, suggesting potential interactions with stimulus representations. Since both movement and stimulus representations are concentrated in the low-dimensional on-manifold subspace (Figs. 3, 4), we hypothesized that such interactions might occur there. Prior work has shown that movement modulates visual responses multiplicatively^1,26^, but Stringer et al. reported that movement and stimulus representations are orthogonal, i.e., exhibit no additive interactions^1^. In particular, their analysis demonstrated that stimulus identity cannot be decoded from the movement-encoding subspace (see Stringer et al., Fig. 4), indicating that stimulus representations do not influence movement representations. Applying a similar analysis to our data, we obtained the same result (Supplementary Fig. S8B). However, a more intriguing question is whether movement representations influence stimulus representations. To address this, we performed the converse analysis, testing whether movements could be predicted from the stimulus-encoding subspace (Fig. 6A), as would be expected if the movement representation extended into that space. We found that face motion could indeed be predicted from the optimal stimulus-encoding subspace (Fig. 6B, left) far better than from a random subspace of equal dimensionality. These movement-stimulus interactions were mediated by the on-manifold subspace (Fig. 6B, right), which is particularly striking given that the on-manifold subspace is lower-dimensional and carries less total movement (Fig. 2D, G) and stimulus (Fig. 4C) information. The interaction we observed was additive and persisted even after removing multiplicative gain modulation from neuronal responses (Supplementary Fig. S8A, see “Methods”). Consistent with Stringer et al.’s published results^1^, we did not observe these additive movement-stimulus interactions in their calcium imaging dataset (Supplementary Fig. S9). We suspect this difference arises because their recordings were from superficial layers, whereas ours were from deep layers, which are affected more strongly by movements^27,28^.

The restriction of movement-stimulus interactions to the on-manifold subspace led us to hypothesize that these interactions are mediated by multiplexing of movement and stimulus representations in chorister neurons. Supporting this idea, choristers exhibited stronger modulation by both movement and stimuli than soloists (Fig. 6C). Moreover, movement and stimulus information were multiplexed within the same choristers, rather than in distinct subpopulations (Fig. 6D). These effects were independent of the size of the time bins (Supplementary Fig. S10). To test whether this multiplexing mediates movement-stimulus interactions, we used GLMs to regress out movement and stimulus modulation from different subpopulations of neurons, then predicted face motion from the full population (see “Methods”). Consistent with our hypothesis, removing movement and stimulus modulation from choristers (top third of population coupling) significantly reduced decoding accuracy, whereas removing it from soloists (bottom third) had no effect (Fig. 6E).

### Off-manifold coding enables stimulus and movement representations to coexist separably in chorister neurons

**Fig. 7:**
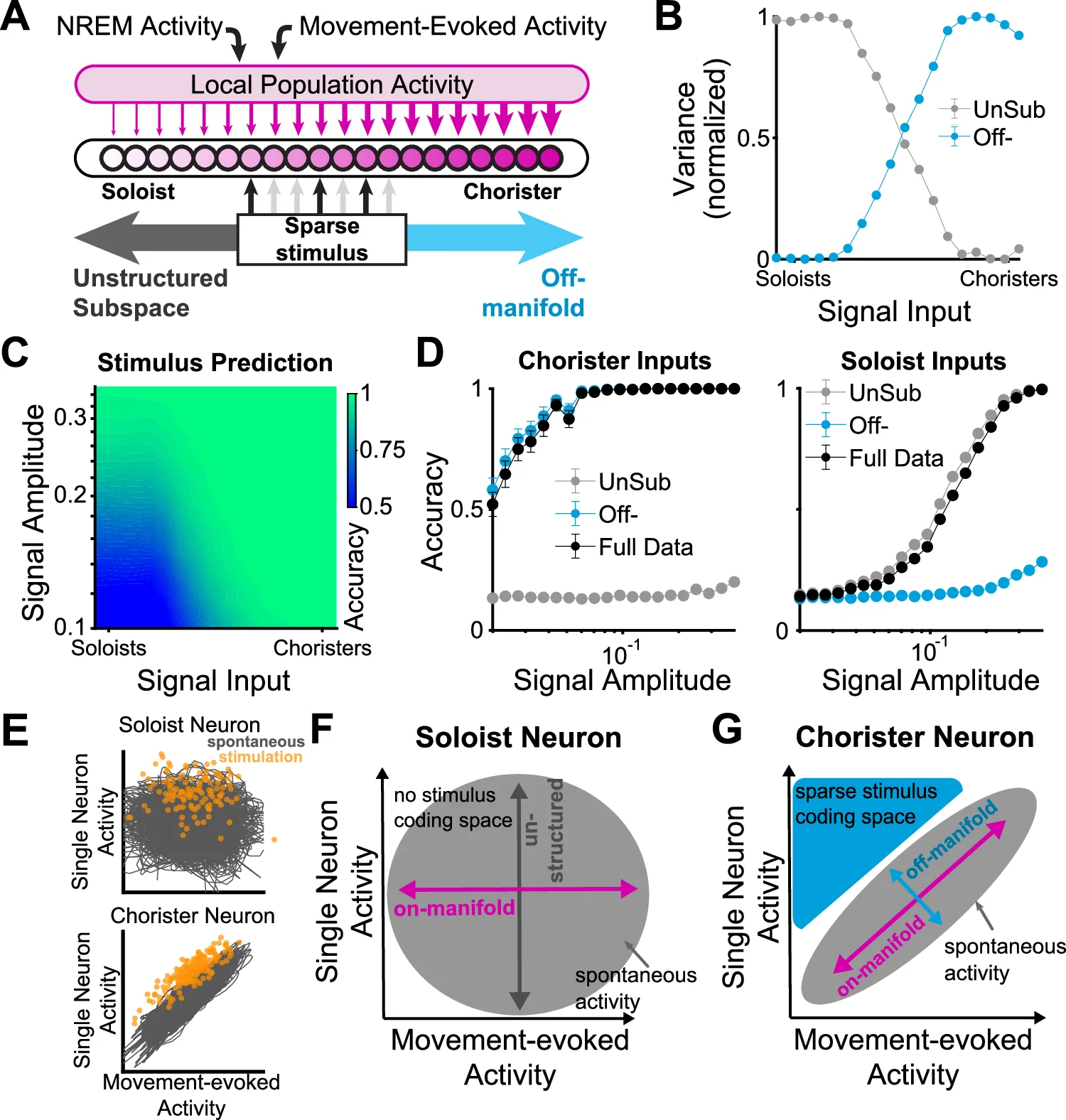
Off-manifold coding enables stimulus and movement representations multiplexed in the same neurons to remain separable. **A** To understand the consequences of multiplexing movement and stimulus representations in the same cells, we used a firing rate model featuring neurons that receive input from the local population with varying strengths to create choristers (strong coupling) and soloists (weak coupling). The local population is modulated by either noise (NREM) or a movement signal (wakefulness). Stimuli are delivered as sparse inputs to a subset of neurons ranging from soloists to choristers. **B** Providing stimulus inputs to soloists creates unstructured subspace coding, but providing stimulus inputs to choristers creates off-manifold coding (*n *= 100 repeats, error bars represent s.e.m.). **C** With sufficiently strong signals, stimuli are equally decodable from soloists or choristers (same axis as **B**). However, with weaker signals, choristers encode stimuli more effectively. **D** This advantage is mediated by off-manifold coding, which retains high decoding accuracy as the signal amplitude decreases (*n *= 10 repeats, error bars represent s.e.m.). **E** (*top*) The model reveals that stimulus-evoked activity (orange) in soloists overlaps with movement-evoked activity (gray) and thus cannot be separated. (*bottom*) In choristers, they are largely non-overlapping and therefore easier to separate (signal amplitude = 0.18). **F** An intuitive explanation for this phenomenon is that soloists are decoupled from the intrinsic manifold, enabling movement-evoked activity to traverse the entire space, leaving no room to encode stimuli without interference. **G** Choristers' coupling to the low-dimensional intrinsic manifold creates a complementary off-manifold subspace (cyan) comprising dimensions suppressed during intrinsic dynamics that movement-evoked activity is unlikely to enter. Population-sparse stimulus-evoked activity accesses this off-manifold coding space, enabling stimuli to be encoded separably from movement-evoked activity.

The result that movement and stimulus representations are multiplexed in choristers is consistent with previous work reporting that choristers exhibit stronger modulation by visual stimuli^23,24^ and explains how movement-stimulus interactions can occur. However, intuition suggests that this coding scheme also increases the likelihood of movement-evoked activity interfering with visual coding. To examine how such representations can be organized to balance integration and segregation at the population level, we built a firing rate model of 100 V1 neurons that receive a dense input from the rest of the V1 population (Fig. 7A), whose activity we modeled as a scalar firing rate variable modulated by random noise during NREM and by movement during wakefulness. We used a distribution of input strengths from the population to the 100 neurons, ranging from low to high, enabling the individual neurons to range from soloists to choristers. All neurons were injected with Gaussian white noise, with the noise amplitude scaled so that all neurons received equal levels of non-stimulus-related input “noise," meaning non-stimulus-related input. Soloists thus received mostly uncorrelated noise, and choristers received mostly correlated noise. Within this model, a set of neurons was chosen to encode visual stimuli ("signal”), with each stimulus activating a different sparse subset of those neurons. We then systematically varied the set of neurons receiving the visual inputs, ranging from soloists to choristers (Fig. 7A), enabling us to adjust how much signal-related variance was in the unstructured vs. off-manifold subspaces (Fig. 7B). We found that both unstructured and off-manifold subspaces coding both showed perfect decoding accuracy if the signal was sufficiently strong (Fig. 7C, top); however, when the signal was weaker, encoding stimuli in choristers was superior (Fig. 7C, bottom). This was attributable to choristers using the off-manifold space (Fig. 7D), similar to what we observed experimentally (Fig. 4C–F). Taken together, these results support the counterintuitive conclusion that stimulus and movement representations can be more easily separable when both are encoded together in choristers than when stimulus representations are encoded separately in soloists.

Our model provides an intuitive explanation for this phenomenon: because soloists’ spontaneous activity is decoupled from population movement-evoked activity, it can traverse the entire state space (Fig. 7E, top). Consequently, there is no distinct region where stimuli can be encoded separately from movements (Fig. 7F). In contrast, choristers’ spontaneous activity is coupled to population movement-evoked activity, confining it to a low-dimensional manifold (Fig. 7E, bottom). This creates an off-manifold portion of state space that non-stimulus-evoked activity rarely enters (Fig. 7F). When stimuli evoke population-sparse activity in choristers, it enables their representations to access this off-manifold subspace, thereby minimizing interference from movement-evoked activity.

## Discussion

In this study, we used NREM sleep to identify intrinsic on- and off-manifold subspaces in deep layers of mouse V1. We found that local circuit dynamics constrain movement-evoked activity by concentrating it in the intrinsic on-manifold subspace, while the fine structure of the representation reflects the specific kinematics of each movement. Visual stimuli are concentrated in both on- and off-manifold subspaces: the on-manifold component mediates low-dimensional additive interactions with movement, whereas the off-manifold component exploits a reserved coding space to store higher-dimensional representations separable from movement representations. Importantly, the observation that stimulus-evoked activity extends beyond the intrinsic manifold is not, in itself, informative; rather, the key result is that intrinsic dynamics define specific suppressed directions that are selectively engaged by stimulus representations. These effects arise from the multiplexing of movement and stimulus representations in chorister neurons, revealing a mechanism by which the brain balances the integration and segregation of movement and sensory representations.

Our finding of additive interactions between movement and stimulus representations contrasts with the results of Stringer et al., who reported no such interaction (see Stringer et al., Fig. 4). Our reanalysis of their dataset (Supplementary Fig. S9) confirmed their results, suggesting that the discrepancy is not due to differences in analysis methods. While differences in recording modality may contribute, we believe the more likely explanation is recording depth: Stringer et al. targeted superficial layers, whereas our recordings were from deep layers. This aligns with growing evidence that movement representations differ between cortical layers^27,28^ and may support distinct population-level computations. We propose that on-manifold movement-stimulus interactions in deep layers could support the integration of self-motion information into predictive coding. This hypothesis predicts that, even in the absence of visual input, V1 should preferentially encode the specific movements expected to most strongly influence retinal inputs. This is supported by recent findings that rodent V1 strongly represents movements of the head and neck^4,29^. It may also partially explain the apparent species difference in which locomotion exerts greater influence over V1 activity in mice^1,2^ than primates^5^ because mice have side-facing eyes, increasing the expected influence of locomotion on retinal inputs. Conversely, V1 activity in primates is strongly influenced by eye movements^6,7^, which are the largest expected source of changes in retinal input. A key limitation of our study is the use of head fixation, which severely constrains movement. Future work examining movement-stimulus interactions in freely moving animals will be important for testing these ideas under more ethologically relevant conditions.

It is widely proposed that intrinsic dynamics serve to restrict neuronal activity to a low-dimensional manifold where relevant computations occur^30–33^. Importantly, these dynamics do not only define dominant activity patterns, but also activity patterns that are suppressed, thereby structuring both the on- and off-manifold subspaces. Our findings suggest that V1 employs this strategy to support low-dimensional interactions between movement and stimulus representations (Fig. 6B). However, visual processing is generally considered the main function of V1, and it is noteworthy that this occurs mostly in the off-manifold subspace (Fig. 4). This suggests that an underappreciated function of low-dimensional intrinsic dynamics may be to reserve a separate off-manifold coding space that can store higher-dimensional representations^34–36^ with minimal interference from movement. Further, our finding that off-manifold coding comprises population-sparse activity in chorister neurons (Fig. 5E–G) reveals an unexpected link between dimensionality, sparse coding, and the chorister-soloist spectrum. Choristers and soloists exhibit anatomical differences suggestive of distinct cell types^23,24^, and our results suggest they also serve distinct computational roles. To clarify a point of potential confusion, the chorister-soloist axis is a stable property of individual neurons related to their anatomical connectivity. In contrast, population sparseness is a transient property of activity patterns across the entire population. In the absence of visual stimuli, choristers will be less likely than soloists to participate in population-sparse activity patterns. However, off-manifold coding refers to stimulus-evoked activity, which drives both choristers and soloists. Stimulus representations are population-sparse in V1 and other brain areas across species^25,35,37,38^, and this has traditionally been interpreted as a mechanism for energy conservation^39^. Our findings suggest a complementary function: sparse coding may allow access to the off-manifold subspace carved out by intrinsic low-dimensional dynamics, thereby minimizing interference from movement-related activity (Fig. 7).

This study has several important limitations. First, we did not use nonlinear dimensionality reduction methods, as existing techniques cannot reliably identify off-manifold structure. Our heuristic for defining on-manifold, off-manifold, and unstructured subspaces is also somewhat arbitrary; while it aligns well with the data in this case (Fig. 4E, F), it may not generalize across conditions or datasets. We also did not identify which specific movement or stimulus features are encoded in the on-/off-manifold or unstructured subspaces, which will require future studies with tasks and stimuli carefully designed to address those specific questions. Perhaps most importantly, we do not yet understand the specific circuit-level mechanisms enabling visual stimuli to evoke more off-manifold activity than movements. In our computational models, we artificially created on/off/unstructured activity by targeting inputs to specific subpopulations of cells (Fig. 7A, B), but this is not intended as a unique or mechanistic explanation. Rather, it serves as a proof of principle that differences in how inputs engage the circuit can give rise to the observed geometry of population activity. In vivo, many possible mechanisms could contribute, including differences in input correlation structure, anatomical targeting of specific cell types or dendritic compartments, and nonlinear circuit operations such as lateral inhibition^40^, coincidence detection^41^, and divisive normalization^42^. These possibilities are not mutually exclusive, and the present data cannot distinguish among them. Disentangling their relative contributions will require large-scale biophysical models constrained by measurements from many experimental modalities, representing an important direction for future work.

More broadly, these results suggest that intrinsic dynamics provide a general organizing framework for population activity, rather than simply constraining it. Our findings also highlight the value of leveraging sleep as a tool to study awake brain function in the visual cortex. While many previous studies have compared stimulus-evoked and awake spontaneous activity in V1^23,26,43–46^, our results suggest that NREM sleep provides a more reliable estimate of intrinsic population structure (Supplementary Fig. S2). Similar approaches have yielded insights in spatial navigation-related circuits^8–10,12^, and we believe this strategy is broadly applicable for studying on- and off-manifold coding in many other brain regions. We encourage others to incorporate sleep recordings into their experiments as a relatively low-effort means to gain insight into the interactions between intrinsic dynamics and representations of behaviorally relevant variables.

## Methods

All experimental procedures were conducted in accordance with the Institutional Animal Care and Use Committee of Albert Einstein College of Medicine.

### Stereotactic surgery and data acquisition

Experiments were conducted using C57BL/6J x FVB F1 hybrid mice^47^. Probes were implanted following similar methods described previously^48^. Briefly, the animals were anesthetized with isoflurane, the skin was prepared for surgery, and a midsagittal cut was made to access the skull. After cleaning the skull, two wires were implanted above the cerebellum for ground and reference. We implanted either a 64-site silicon probe (*n *= 3 mice with NeuroNexus Buzsaki-5 × 12-64; *n *= 1 mice with F-series Cambridge Neurotech) or 128-site silicon probe (*n *= 1 mouse with 2 silicon probes 64-site F-series Cambridge NeuroTech stacked) in the primary visual cortex (V1) (AP: 3.4 ML: 2.6 DV: 0.6 from brain surface), for a total of *n *= 5 mice, and a copper mesh hat was built to shield the probes. Probes were previously mounted on microdrives that were used to move the probe farther into V1 for maximizing unit yield, though never beyond a DV of 1.0 mm. In a subset of animals (*n *= 3 mice), we also injected adeno-associated viral vector (AAV) with a CaMKII promoter to express channelrhodopsin-2 in pyramidal cells in the primary somatosensory (S1) and primary motor (M1) cortex. The recombinant AAV vector was pseudotyped with an AAV5 capsid and packaged by Addgene. The coordinates used were the following: S1 (2 injections: (1) anterior-posterior (AP): − 1.5 mm, medial-lateral (ML): 1.5 mm, Depth (D): 0.4 mm; (2) D: 0.8 mm) and M1 (2 injections: (1) AP: 1.5 mm, ML: 1.7 mm, D: 0.75 mm; (2) D: 1.5 mm). Each injection site received 500 nL of undiluted AAV solution. After three weeks, we implanted two optical fibers (400 μm core diameter, 0.5 NA) into the left hemisphere, targeting S1 (AP: − 1.5 mm, ML: 1.5 mm, D: 0.6 mm) and M1 (AP: 1.5 mm, ML: 1.7 mm, D: 1.25 mm). Optogenetic stimulation was at a wavelength of 450 nm (Osram PL450B laser diode). Animals were housed individually after implantation and allowed to recover for at least one week before experiments. Recordings were performed using the Intan system at 30 kHz. Offline automatic spike sorting was performed using Kilosort 2.0^49^, and all parameters used for sorting are presented in the Kilosort 2.0 wrapper repository in StandardConfig_KS2Wrapper.m. Manual curation was performed using Phy, our recordings yielded an average of 160 units per session. Isolated single units were classified as putative principal neurons or fast-spiking interneurons based on trough-to-peak time of waveforms, a criterion previously used in V1^50^.

### Visual stimuli

The natural scene stimuli were the same dataset used in ref. ^1^. Briefly, a total of 32 images were selected from ethologically relevant categories, such as animals and plants. The images chosen were less than 50% uniform background, and with a balance of low and high spatial frequencies. Each stimulus was presented for a 0.5 s duration, and the inter-stimulus interval varied between 0.7 and 1.0 s. Stimuli were presented on a screen facing the eye contralateral to the side being recorded, with 60 repetitions of each image.

### Optogenetic playback of running speed

For the optogenetic stimulation waveform, we used the mouse’s own running speed, which was tracked using a treadmill integrated into the head-fixation recording rig. From the baseline head-fixation period, we manually selected a 5 s window that contained running speed activity to be played back in the optical fibers of M1 and S1. This waveform was then scaled to one of the three intensities used (1 mW, 1.5 mW, and 2.1 mW on average) and played 20 times in each optical fiber in an alternating fashion, for a total of 60 stimulations. We used a 5 s interval between each stimulation.

### Sleep state detection

We used an automated state scoring algorithm to detect sleep and awake states; this algorithm was previously described in greater detail^51^, and it is available in Buzcode. The automatic scoring was manually inspected and corrected when necessary. In the automatic state scoring, the spectrogram of LFPs was calculated, and PCA was performed on the spectrograms. The first PC reflects the activity in lower frequencies, so a threshold was used to define periods of Non-REM (NREM), i.e., high PC1 power. The lower PC1 power periods were then subdivided into awake and REM states. For that, a narrow band theta power ratio (5-10 Hz/2-16 Hz) and EMG estimations were used to identify REM (low EMG activity and high theta power ratio). The EMG activity was estimated from the LFP high-frequency coherence across all recording channels. We only used periods of freely moving recordings for state scoring, not including the head-fixation periods. All of our analyses in awake and NREM states used the periods identified by this detection.

### Face motion recording and extraction

We used an infrared camera (FLIR model number BFLY-U3-23S6M-C USB 3.0 Blackfly, mounted with an infrared lens) pointed at the mouse’s face to record movements. Videos were collected at 30 Hz and aligned with electrophysiology based on digital pulses that triggered frame collection. To extract the face motion principal components, we used the SVD computation version of Facemap^1,20^; In brief, the absolute motion energy is extracted as the absolute value of the difference between frames, subsequently, a singular value decomposition (SVD) is applied to the absolute motion energy, this method yields 500 face motion PCs that were used in our analysis. We excluded ROIs around the eyes to avoid contamination in the face motion PCs due to changes in pupil diameter.

### Data analysis

We used custom MATLAB and Python scripts for analysis and plotting. In our analysis, we used the following toolboxes: Buzcode, communication subspace^52^ for Reduced Rank Regression, GPML toolbox for Gaussian Process Regression, and Pynapple^53^. We only used recording sessions containing a minimum of 30 neurons and 20 min of sleep, and for analyses utilizing the off-manifold subspace, we used a minimum of 50 neurons. When analyzing running speed and face motion prediction, we excluded sessions where the prediction from the full data was smaller than a threshold of *R*^2 ^= 0.1. In the classification of natural visual scenes, we excluded sessions in which the full data prediction F1 score was below 35%.

### Cross-validated PCA

We used cross-validation of principal component (PC) dimensions to determine the variance explained by each dimension in NREM neuronal data. In our analysis, we first binned the spike times of each unit in 0.1 s bin sizes without overlap or smoothing. Next, we split the data into 5-fold training and test sets, *N*_train_ and *N*_test_, respectively. To find the PCs (*V*_*n*_, where *n* is the principal component index), we performed singular value decomposition on the z-scored training set (*N*_train_). Next, we projected the z-scored test set data (*N*_test_) onto the PCs to obtain cross-validated scores *U*_*n*_ = *N*_test_*V*_*n*_. The eigenspectrum was estimated by the variance of each cross-validated PC (cvPC), which was calculated by the variance of each score *U*_*n*_.

#### Empirical eigenspectrum null distribution and subspace identification

We defined the eigenspectrum null distribution based on a variant of Horn’s parallel analysis^54^. We shuffled the cell identities of the test set (denoted as *N*_test-shuffled_) and estimated the null scores by projecting the shuffled data onto the original PCs, from the intact training set, as in *U*_*n*-null_ = *N*_test-shuffled_*V*_*n*_. The null eigenspectrum is then the variance identified in each null score *U*_*n*-null_. This procedure is repeated 10,000 times so we can build a confidence interval (CI) of the null eigenspectrum. We defined the CI as the lower (100 × *p*/2)% and upper (100 × (1 − *p*/2))% values of the null eigenspectrum, where *p* = 0.05 and *N* is the number of units in the data. The CI was used to define where on-/off-manifold and unstructured subspaces begin and end in the cross-validated eigenspectrum. Their definition is as follows: (1) on-manifold: cvPCs with variance above the upper CI bound of the null, (2) unstructured subspace: cvPCs with variance within the upper and lower null CI bounds, (3) off-manifold: cvPCs with variance below the lower null CI bound.

### Subspace alignment

To measure the overlap of explained variance between awake and NREM subspaces, we use the subspace alignment index introduced in ref. ^55^. In brief,^55^ introduces a metric to estimate how much variance the PCs of one state (state 2) explain in the data of another state (state 1). The subspace alignment is shown in (1)

1 $${\rm{Subspace}}\,{\rm{Alignment}}=\frac{{\rm{Tr}}({{\bf{V}}}_{s2}^{\top }{{\rm{Cov}}}_{s1}{{\bf{V}}}_{s2})}{{\rm{Tr}}({{\bf{V}}}_{s1}^{\top }{{\rm{Cov}}}_{s1}{{\bf{V}}}_{s1})}$$

Where Cov_*s*1_ is the covariance matrix during state 1, *V*_*s*2_ is the matrix with the top PCs found during state 2, and *V*_*s*1_ is the matrix of top PCs from state 1, whereas ${\rm{Tr}}()$ indicates the matrix trace. This metric varies from zero to one, with one indicating the highest subspace alignment. We cross-validated the subspace alignment by splitting the data of state 1 into two folds, one for training and the other for testing, such that the *V*_*s*1_ is calculated in the training set, and Cov_*s*1_ is determined in the testing set. The cross-validating procedure can lead to a subspace alignment larger than one. The **[1]** vector was regressed out of both states, thus not influencing the alignment metric (see *Removing global firing rate fluctuations* section). For the subspace alignment comparison between awake and NREM PCs, state 1 was designated as the awake state, and state 2 as the NREM state.

### Random subspaces

Each NREM subspace (on-/off-manifold and unstructured subspace) contained a different number of dimensions. The additional orthogonal dimensions could carry more information, complicating direct comparisons of prediction performance between subspaces. To control for this, we generated random orthogonal subspaces with equal dimensionality as the on-/off-manifold and unstructured subspaces. The random subspaces were generated by calculating the singular value decomposition (SVD) of Gaussian random matrices with size (*N*_*neurons*_ × *N*_*neurons*_) and using the right singular vectors (*V*_*random*_). The number of selected random vectors was adjusted to match the dimensionality of sleep subspaces (on-/off-manifold and unstructured subspaces). This procedure was repeated 10 times for each subspace, unless otherwise stated, to average chance fluctuations in the random subspaces. For each repetition, we generated a new random matrix to draw *V*_*random*_.

#### Prediction index

To assess whether NREM subspaces (on-/off-manifold and unstructured) predicted variables better than random orthogonal subspaces, we compared the prediction performances using the Prediction Index (8).

### Face motion prediction from neuronal activity

We used Reduced Rank Regression (RRR) to predict face motion PCs activity from neuronal activity. We used face motion activity from the first 40 min of head fixation, which were free of both visual stimulation and optoplayback. The face motion PCs were downsampled to bins of 100 ms before the prediction. The neuronal firing rate was calculated over the same binning window; the rates were then z-scored before training or testing the model. We used the same implementation of RRR as described in ref. ^52^. Briefly, this method initializes the *β*’s used for the RRR with ridge regression, using cross-validation parameter selection for the ridge parameter (*λ*), RRR is then calculated based on the *β* from ridge regression. The optimal rank for prediction was selected based on peak performance on cross-validated *R*^2^.

### Running speed and optoplayback prediction from neuronal activity

We used Gaussian Process Regression (GPR) for predicting running speed and optoplayback from neuronal activity. Running speed and optoplayback were downsampled to 100 ms for prediction. Running speed was further log-transformed before model training and prediction. Neuronal firing rate was calculated over the same 100 ms binning window, and the rates were z-scored before training or testing the model. We used the GPML MATLAB toolbox (available at: https://gaussianprocess.org/gpml/code/matlab/doc/) for the fitting.

A Gaussian Process is defined as:

$$f({\bf{x}}) \sim {\mathcal{GP}}\left(m({\bf{x}}),k({\bf{x}},{{\bf{x}}}^{{\prime} })\right)$$

Where *m*(**x**) is the mean function, and $k({\bf{x}},{{\bf{x}}}^{{\prime} })$ is the covariance function (kernel). We fit the GPR with *m*(**x**) = 0, assuming no prior bias in the data, and with the covariance function Rational Quadratic Kernel, defined as:

2 $${k}_{{\rm{RQ}}}({\bf{x}},{{\bf{x}}}^{{\prime} })={\sigma }_{f}^{2}{\left(1+\frac{\parallel {\bf{x}}-{{\bf{x}}}^{{\prime} }{\parallel }^{2}}{2\alpha {\ell }^{2}}\right)}^{-\alpha }$$

Where ${\sigma }_{f}^{2}$ is the output variance, *ℓ* is the characteristic length-scale, and *α* is the shape parameter. We optimized the hyperparameters by maximizing the marginal likelihood, and they were initialized as zero. We used a Gaussian likelihood for the inference of the posterior process (regression).

Our choice of GPR over more interpretable models (such as GLMs) is based on GPR’s improved prediction performance for these variables compared to other models, allowing for the inclusion of more recording sessions with meaningful prediction performance in the analysis.

### Recurrent Neural Network model of feedforward input

To test whether feedforward inputs to a neural network could occupy overlapping subspaces, we created a recurrent neural network rate model with two distinct feedforward inputs.

The network consisted of 100 neurons operating at time step *Δt* = 0.01*s*, connected by the recurrent weight matrix *J*. We initialized *J* using a scaled normal distribution given by ${{\bf{J}}}_{ij} \sim {\mathcal{N}}(0,{g}^{2}/{N}_{{\rm{neurons}}}),\mathrm{with}\,g=1.8$, and set all self-connections (*i* = *j*) to zero. To ensure an identifiable separation of neurons in the intrinsic manifold, we applied a gradient-based population coupling mask to the inputs of *J*, which linearly increased from 0 to 1 across neurons, while maintaining the outputs intact. This created a group of neurons that receive a small or no input from other neurons (low population coupling), while the others receive input from all other neurons (high population coupling). This facilitates access to the intrinsic manifold subspace by targeting the neurons that feed into the highly coupled neurons. Neuron currents had a decay time constant *τ* = 0.05*s* and independent noise *η* drawn from a Gaussian distribution, with the amplitude of the noise scaled by the inverse of the population coupling mask (*A*_*noise*_ = 1 − Mask_population coupling_), so that the independent noise of a neuron with low population coupling is relatively higher than that of neurons with high population coupling.

Feedforward inputs (*I*_*FF*,1_and*I*_*FF*,2_) were modeled as two sine waves of frequency 0.5 Hz at 45.8 degrees out of phase and enveloped so that the feedforward inputs never overlap in time. The envelope for each input was created as a sine wave of 0.05 Hz and 180 degrees out of phase; any values below zero were set to zero, and any values above zero were set to 1 to create all-or-none envelopes. The feedforward input weights (*W*_*FF*,1_and*W*_*FF*,2_) targeted two randomly selected neurons within the interval [0, 30], consisting of neurons with low population coupling. Each weight was drawn from a uniform distribution in the interval [0, 1), and normalized by its *ℓ*_2_-norm. The input amplitude was set to 0.3. The feedforward input simulation was run for 100 s.

To define on-/off-manifold and unstructured subspaces, we first simulated a 400 s baseline period without feedforward inputs to simulate sleep activity, and then we defined the subspaces using cross-validated PCA, as in real data.

The model can be summarized by the following equation (3):

3 $$\tau \frac{{\rm{d}}{x}_{j}}{{\rm{d}}t}=-{x}_{j}+{\bf{J}}{\bf{r}}+{{\bf{W}}}_{FF,1}{{\bf{I}}}_{FF,1}+{{\bf{W}}}_{FF,2}{{\bf{I}}}_{FF,2}+\eta$$

### Optoplayback neuronal subspace

We used partial least squares (PLS) regression to identify the neuronal subspace associated with the optoplayback of running speed. We downsampled the optoplayback waveform to 100 ms and focused on the 6 s stimulation epoch, which included the 5 s stimulation window, as well as an additional 0.5 s before and after the stimulus onset and offset. All the epochs were then concatenated in an array for regression. Neuronal firing rates were calculated over the same 100 ms binning window. Rates were z-scored before training and testing the model.

PLS regression identifies dimensions in neuronal activity space **X** that maximize the prediction of the optoplayback waveform (*y*). The commonly used SIMPLS algorithm^56^, works by iteratively extracting these dimensions. The algorithm then deflates **X** and *y* by regressing out the PLS dimensions, and the process is repeated until all the dimensions are identified.

The standard SIMPLS algorithm does not guarantee orthonormal loadings; therefore, we modified the algorithm to achieve this. We changed the algorithm to operate in the PCA space (*V*_*x*_) of **X** instead of directly in the neuronal space. After extracting each PLS dimension *w*_*i*_, we regress out *w*_*i*_ from *V*_*x*_ and perform the singular value decomposition on the resulting matrix. We then retain the top *N* − *i* left singular vectors, where *N* is the rank of **X** and *i* is the i-th iteration of PLS regression. This process ensures orthonormal loadings while preserving the relationship between neuronal activity and the optoplayback waveform.

To determine the number of PLS dimensions in the optoplayback subspace, we used cross-validated *R*^2^ to identify the number of cumulative dimensions that reach peak performance. To avoid individual validation folds dominating the estimate, we normalized per-fold *R*^2^ values (scaling them to a 0-1 range) and selected the number of dimensions corresponding to the peak in average cross-validated *R*^2^, ensuring that dimensionality selection is based on consistent performance across all folds.

#### Orthogonality measure using cross-subspace prediction

Testing orthogonality in high-dimensional spaces can be ambiguous because in a large enough space, any random vector can be orthogonal to any other vector. To assess whether two subspaces overlap or interact, we employed a cross-subspace prediction framework.

For each subspace comparison, we used the binned neuronal firing rate (*N*) and the target variable (*y*) split into training and test sets (*N*_train_ and *N*_test_, *y*_train_ and *y*_test_, respectively). Both the train and test sets of neuronal firing rate were z-scored. On the training set, we trained a predictive model to map the neuronal data to the targeted variable, as in *g*(*N*_train_, *y*_train_), where *g* represents the predictive model. We also use the training set to identify the subspace (*V*) to be tested, if the subspace is contained within the same data time period. In the test set, we reduced the neuronal firing rate data to the subspace, as in *N*_reduced_ = *N*_test_*VV*^⊤^, before inputting into the model to predict the target variable (${\hat{Y}}_{{\rm{test}}}=g({N}_{{\rm{reduced}}})$), we then compared the prediction performance to *y*_test_. To control for spurious subspace alignment, we also created random subspaces with orthogonal vectors (*V*_rand_) and repeated the procedure using these random subspaces. We then compare the performance of the tested subspace with the random subspace. A statistically significant difference indicated subspace overlap or interaction.

In Fig. 3, we use this method to compare the orthogonality of the following: RNN activity during feedforward input 1 and 2, the V1 neuronal firing rate optoplayback versus running speed, face motion, and visual stimulation. In Fig. 6, we used this method to compare the orthogonality of face motion with the visual stimulation subspace, where their dimensionality is matched based on which subspace is smaller. For all these comparisons, the [**1**] vector was regressed out of the data, thus not influencing the subspace overlap. See the “Removing global firing rate fluctuations" section for more details.

### Natural visual scenes classification

We predicted stimulus identity by fitting an unnormalized multiclass linear discriminant analysis model (LDA). We calculated the average firing rate of each neuron during a single stimulation window, which was repeated for every scene stimulation and its repetitions. In every analysis, the firing rate of each neuron across all stimulus presentations was z-scored and used as a predictor variable. We projected the neuronal data into different subspaces (like on-/off-manifold and unstructured subspaces) for some of the analyses in this study.

In our unnormalized LDA, the neuronal data (*D*) is projected into dimensions that maximize differences between labels, which we denote as *D*_*bc*_. To achieve this, we first computed the average firing rate for each stimulus identity and subtracted the overall mean response across stimuli. We then performed PCA on *D*_*bc*_, which returns *p* − 1 informative dimensions; we therefore used the top 31 principal components. We refer to this approach as unnormalized LDA because the PCA step is applied solely to maximize inter-class separation, without the normalization used in standard LDA to minimize within-class variance. Since much of the within-class variance arises from movement-evoked activity, standard LDA artificially orthogonalizes the stimulus- and movement-predictive subspaces. For classification, we trained a Naive Bayes classifier in this unnormalized LDA space and applied the Maximum A Posteriori (MAP) rule to assign each stimulus to the class with the highest posterior probability.

#### Visual stimulation subspace

To find the visual stimulation subspace, we employed the same approach as the unnormalized LDA, but without the classification step. If the neuronal data *D*_*bc*_ represents the average firing rate matrix of size *N*_neurons_ x 32, where 32 is the number of stimuli, and the average across all stimuli is removed from this matrix, the visual stimulation subspace is given by the top 31 principal components of *D*_*bc*_

In some analyses, this subspace was matched in dimensionality with movement subspaces for comparison. In that case, only the top-ranking dimensions are kept.

#### Movement gain modulations

To control for movement-related modulations of visual responses, we fitted a model that captured both multiplicative and additive effects of movement on neuronal activity. Neuronal responses *r*_*n*_(*t*) of neuron *n* at time *t* were modeled as:

4 $${r}_{n}(t)={f}_{n}({S}_{t})\left(1+{{\boldsymbol{\beta }}}_{n}^{\top }{{\bf{m}}}_{t}\right)+{{\boldsymbol{\alpha }}}_{n}^{\top }{{\bf{m}}}_{t}+{\epsilon }_{t},$$

where *f*_*n*_(*S*_*t*_) represents the stimulus-driven response to stimulus *S*_*t*_, **m**_*t*_ is a vector of face motion PCs activity at time *t*, ***β***_*n*_ and ***α***_*n*_ are neuron-specific coefficients capturing multiplicative and additive modulation gain by face movements, respectively, and *ϵ*_*t*_ is residual noise. We also fitted multiplicative and additive gain modulation alone to investigate them in isolation.

The models were fit using ordinary least squares regression with an alternating optimization procedure, where we fix *f*_*n*_(*S*_*t*_) to estimate ***β***_*n*_ and ***α***_*n*_, and then update *f*_*n*_(*S*_*t*_) while holding the movement parameters fixed. These steps are repeated until convergence.

We performed *k*-fold cross-validation to assess model performance and identified neurons for which the movement modulation parameters explained a significant fraction of variance (mean cross-validated *R*^2^ > 0). For these neurons, we removed movement-related effects from the neuronal responses by subtracting the predicted movement-dependent components:

5 $${r}_{n}^{\,\mathrm{resid}\,}(t)={r}_{n}(t)-{f}_{n}({S}_{t}){{\boldsymbol{\beta }}}_{n}^{\top }{{\bf{m}}}_{t}-{{\boldsymbol{\alpha }}}_{n}^{\top }{{\bf{m}}}_{t}.$$

The resulting residuals ${r}_{n}^{\,\mathrm{resid}\,}(t)$ were used in subsequent analyses to focus on purely stimulus-driven representations. In all analyses, we used only the first face motion PC since we did not see a substantial improvement in prediction performance as we added more movement PCs.

### *R*^2^ and F-1 score

To estimate the performance of regression analyses in face motion, running speed, and optoplayback, we use the equation (6) for *R*^2^.

6 $${R}^{2}=1-\frac{\sum\limits_{i}{({y}_{i}-\hat{{y}_{i}})}^{2}}{\sum \limits_{i}{({y}_{i}-\bar{y})}^{2}}$$

Where *y* is the original variable, $\hat{y}$ is the model predicted and $\bar{y}$ is the mean of the original variable. In this *R*^2^ equation, the model is compared to a constant model (the mean of the original variable). If the prediction is worse than the constant model, the *R*^2^ can be negative, which is comparable to *R*^2^ = 0. The performance of classification for natural scenes was calculated using the F1-score equation (7).

7 $${\rm{F}}1\,{\rm{score}}=2\cdot \frac{({\rm{Precision}}\cdot {\rm{Recall}})}{({\rm{Precision}}+{\rm{Recall}})}$$

Where $\,\mathrm{Precision}=\frac{\mathrm{TP}}{\mathrm{TP}+\mathrm{FP}\,}$ and $\,\mathrm{Recall}=\frac{\mathrm{TP}}{\mathrm{TP}+\mathrm{FN}\,}$, with TP, FP, and FN referring to true positives, false positives, and false negatives, respectively. We cross-validated all the *R*^2^ and F1-score presented in this study. For that, we split the data into five contiguous blocks, with four blocks used for training models and measuring their performance on the one block of held-out data.

8 $${\rm{Prediction}}\,{\rm{Index}}=\frac{{R}_{s}^{2}-{R}_{rp}^{2}}{{R}_{s}^{2}+{R}_{rp}^{2}}$$

Where ${R}_{s}^{2}$ is the *R*^2^ for one the NREM subspaces and ${R}_{rp}^{2}$ is the *R*^2^ for the random subspace. A positive prediction index means the prediction was better in the NREM subspace, and a negative index means the prediction was better in the random subspace. To ensure that the Prediction Index is bounded to the interval [ − 1, 1], we set to zero every *R*^2^ that was negative.

### Reliability index

We quantified the reliability of each PC using the inverse of the coefficient of variation. First, for both awake and NREM states, data were split into five consecutive blocks of equal duration (folds). In each iteration, one fold was used as a training set to compute the PCs, and the other four folds were used to estimate the eigenspectrum, with each fold generating an instance of cross-validated eigenspectrum. This process was repeated so that each fold was used once for training, returning 20 cross-validated eigenspectra in total. Figure S2A shows an example of the average eigenspectrum and the confidence interval built for the 20 cross-validated eigenspectra. Second, for each PC, we computed the mean (*μ*) and the standard deviation (*σ*) across the total 20 cross-validated eigenspectra. Reliability was defined as $\frac{\mu }{\sigma }$. This returns one reliability value per PC, for each state (awake and NREM). The reliability index is a metric to compare the reliability observed in awake versus NREM. It is determined by (9)

9 $${\rm{Reliability}}\,{\rm{Index}}=\frac{({\mathrm{Rel}}_{{\rm{NREM}}}-{\mathrm{Rel}}_{{\rm{Awake}}})}{({\mathrm{Rel}}_{{\rm{NREM}}}+{\mathrm{Rel}}_{{\rm{Awake}}})}$$

Where Rel_NREM_ and Rel_Awake_ is the reliability of a single PC in NREM and awake states, respectively. To summarize the reliability across all PCs in a recording session, we average the reliability index across the entire eigenspectrum. Supplementary Fig. S2B shows an example of reliability for each PC for a single session, while Supplementary Fig. S2C shows the reliability index across multiple sessions.

### Single-neuron population coupling to determine choristers and soloists

Population coupling was calculated as described previously^23,57^; in brief, we summed the neuronal activity of *N* − 1 neurons binned and zero-centered, then took the dot product of the left-out neuron’s activity with that of the summed population activity. The resulting number is normalized by the *l*^2^-norm of the left-out neuron’s activity. For within-session comparison, we normalized the population coupling to the range [0, 1]. When pooling neurons from different sessions, we performed a normalization to the spike shuffling model as described previously^23,57^. Unlike Okun et al., we did not smooth the data before the population coupling calculation. Importantly, we calculated population coupling during NREM, not awake spontaneous activity. This yielded qualitatively similar results (not shown) but avoids the risk of population coupling estimates being biased by movement-evoked activity.

### Population sparseness

We used a previously described population sparseness metric to determine the subspace population sparseness^58^. In brief, if *L*_*i*_ denotes the absolute value of neuron *i*’s loading on a single cvPC, the population sparseness was calculated as:(10)

10 $${\rm{Population}}\,{\rm{Sparseness}}=1-\frac{{\left[\mathop{\sum }\nolimits_{i=1}^{N}\frac{{L}_{i}}{N}\right]}^{2}}{\mathop{\sum }\nolimits_{i=1}^{N}\frac{{L}_{i}^{2}}{N}}$$

This metric ranges from 0 to 1, where a value close to 1 corresponds to high population sparseness. The subspace population sparseness was the average taken from every cvPC belonging to that subspace.

### Single neuron subspace participation

To determine the participation of a neuron in a specific subspace, we calculated the average absolute PC loading for that neuron across all the cvPCs that span that specific subspace.

### Chorister-Soloist subspace index

To assess whether activity in a subspace is primarily composed of choristers or soloists, we first calculated the population coupling for all the cells and defined the choristers to be the neurons with the top 30% coupling, and the soloists to be the bottom 30%. For each subspace (on-/off-manifold and unstructured), we calculated the subspace participation of choristers and soloists. Using these values, we define the Chorister-Soloist subspace index as (11)

11 $$\begin{array}{c}{\rm{Chorister}}/{\rm{Soloist}} \, {\rm{Subspace}} \, {\rm{Index}}=\frac{\left({\rm{Participation}}_{{\rm{choristers}}}-{\mathrm{Participation}}_{{\rm{soloists}}}\right)}{\left({\rm{Participation}}_{{\rm{choristers}}}+{\rm{Participation}}_{{\rm{soloists}}}\right)}\end{array}$$

Where Participation_choristers_ and Participation_soloists_ are the subspace participation of choristers and soloists, respectively. We included both single-unit and multi-unit activity in our calculation of the population firing rate, but the index was only calculated for well-isolated single units.

### Removing global firing rate fluctuations

To control for population firing rate fluctuations, we removed them from the data when applicable. We first generated a vector with the same number of rows as the number of neurons, where each neuron has equal loadings. We call it the **[1]** vector, which contains ${[11...1]}^{\top }\in {{\mathbb{R}}}^{n}$ where *n* is the number of neurons. Next, we get the projection of the z-scored neuronal data (*D*_*t*×*n*_, where *t* is the number of time bins) onto the normalized **[1]**_*n*×1_ vector to estimate the overall firing rate fluctuation in time FR_*t*×1_. Finally, we remove this component from the data as in ${D}_{t\times n}-{\mathrm{FR}}_{t\times 1}\cdot {{\bf{[1]}}}_{n\times 1}^{\top }$

### Up and down state detection

To control for up and down transitions in NREM affecting our estimates of population activity, we detected up/down states and restricted the analysis to up states alone. For detecting these states, we used the methodology described previously^59^. Briefly, during periods of NREM, the LFP delta (0.5–8 Hz) and gamma bands (30–600 Hz) are used for thresholding and detection of up states (high gamma power) and down states (delta peak and low gamma power). Down states are then further restricted to periods where spiking activity is below the threshold.

### Spike subsampling to account for firing rate differences

To prevent differences in baseline firing rates across neurons from influencing our results, we z-scored the binned spike trains in all analyses in this study. However, to account for the possibility that this is inadequate, we also performed a spike subsampling analysis (Supplementary Figs. S2, S9). First, we sorted all neurons by their firing rate and randomly paired each high-firing rate neuron with a low-firing rate neuron. We then subsampled the spike train of each high-firing neuron to contain the same number of spikes as its matched low-firing partner. These spike trains were then binned and normalized using z-scores.

### Movement and visual stimuli multiplexing model

We built a simple neural network to investigate how the overlap of both movement-evoked activity and visual stimuli influences the decoding of the visual stimulus identity. The network consists of 100 principal neurons that are not connected to one another. These neurons received three inputs: a diffuse movement signal, sparse stimulus signals, and their individual uncorrelated noise. The movement-evoked inputs had a weight matrix *W*_*m*_, which was initialized with a normal distribution *N*(0.3, 1) and then normalized to the range [0, 1]. Because this activity was the factor that defined whether the neuron was a chorister or soloist, we zeroed the bottom 50 weights to create 50 soloists. The sparse stimuli were 10 stimuli, each with different loadings. In each interaction, 30 out of 100 neurons received the sparse stimuli. We selected 5 out of 30 to receive all 10 stimuli, and different combinations of 5 out of 25 neurons were chosen for the visual stimulus input. This allowed us to have 10 different combinations of neurons tuned to each individual stimulus. The weights *W*_*s*_ were initialized with a normal distribution *N*(0, 2) and then limited to the range [ − 1, 1] by selecting loadings above or below that value and limiting it to the range. The magnitude of individual uncorrelated noise was input to the cell as a function of their loading in *W*_*m*_, such that *W*_*i*_ = 1 − *W*_*m*_. Thus, choristers have less uncorrelated noise than soloists. The model had a period without the sparse stimuli, which we refer to as the sleep epoch, where we could define on-/off-manifold and unstructured subspaces. During the sleep epoch, only individual noise (*I*_*i*_) and movement-evoked activity (*I*_*m*_) were given as input into the neurons. We then have an epoch of stimulation where we give the sparse stimuli (*I*_*s*_) together with *I*_*i*_ and *I*_*m*_. Each stimulation identity was repeated 10 times. Our model can be summarized by the following equation (12)

12 $$\tau \frac{{\rm{d}}{x}_{j}}{{\rm{d}}t}=-{x}_{j}+{{\bf{W}}}_{m}{{\bf{I}}}_{m}+{{\bf{W}}}_{s}{{\bf{I}}}_{s}+{{\bf{W}}}_{i}{{\bf{I}}}_{i}$$

This model was run with a decay time (*τ*) of 0.1 s, and in time steps (*dt*) of 0.1 s. Sleep and stimulation epochs lasted 200 s each. We ran 100 simulations of this model for the accuracy estimation, and 10 simulations for unstructured and off-manifold subspace accuracy.

### Null hypothesis testing

Information on statistical tests can be found in Supplementary Table S1. All t-tests were two-tailed, and for multiple pairwise group comparisons, we used the Tukey-Kramer post hoc test correction, unless otherwise stated.

### Reporting summary

Further information on research design is available in the Nature Portfolio Reporting Summary linked to this article.

## Supplementary information

Supplementary Information

Peer Review file

Reporting Summary

## Data Availability

The datasets generated and/or analyzed during the current study have been deposited in the Zenodo database under accession code 20013296.

## Code availability

The code used to reproduce the analyses presented in this manuscript is publicly available on GitHub at: https://github.com/SjulsonLab/deOliveira_Kim_SleepV1_2026. An archived version of the repository is also available on Zenodo at: 10.5281/zenodo.20024211.
