# Supplementary material for: Sleep reveals dynamics integrating and segregating movement and stimulus representations in V1
Source: Nat Commun. 2026 Jul 21;17:8911. doi: 10.1038/s41467-026-75347-4 (PMC13503742; doi:10.1038/s41467-026-75347-4)
Supplement: Supplementary file 1 [file 41467_2026_75347_MOESM1_ESM.pdf]

Supplementary Figures

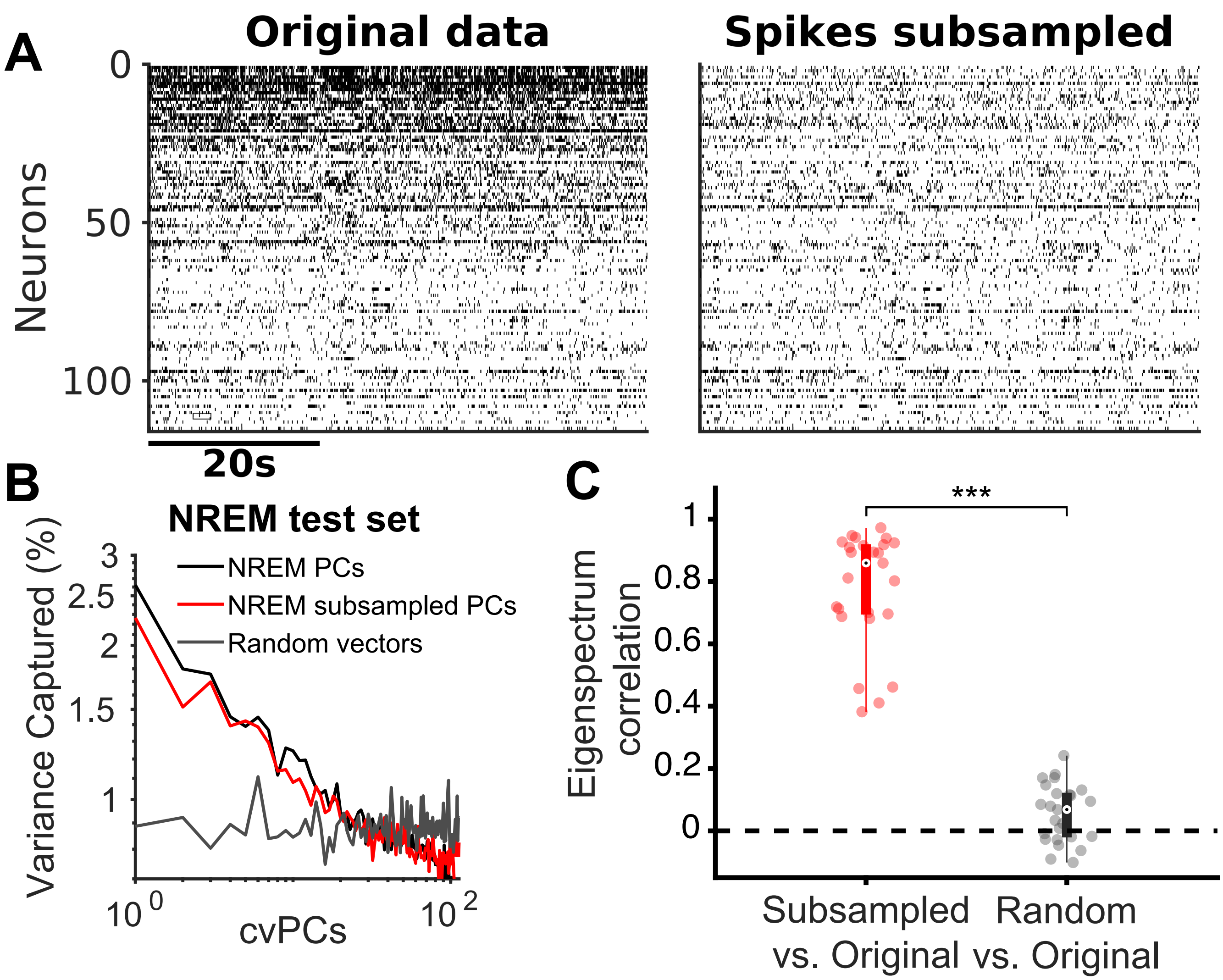

Fig. S 1. The population activity structure observed during NREM is not explained by high firing rate neurons dominating on-manifold dimensions. Binned spike trains are z-scored in every analysis in the main text. However, to address the possibility that this was insufficient to account for differences in firing rate, we also performed a spike subsampling analysis. (**A**) We subsampled the spikes of the top half of high-firing-rate neurons to match the firing rates of the bottom half. The subsampled spike trains were then z-scored prior to cvPCA.(**B**) Example eigenspectrum of original NREM test set projected onto PCs from NREM training set, subsampled NREM training set, and a random subspace. Note the similarity of subsampled and full eigenspectra. (**C**) The NREM PC eigenspectrum is strongly correlated with the subsampled NREM PC eigenspectrum (red boxplot) but not a random subspace (gray boxplot). Correlation between original and subsampled NREM eigenspectrum: 0.78 ± 0.04, random subspace: 0.05 ± 0.02, mean ± s.e.m., n = 25 V1 recording sessions [paired t-test, two-sided, p= 5.27×10^{-16}, ***p<0.001] All box plots show median, 25th–75th percentiles, and 1.5× IQR whiskers.

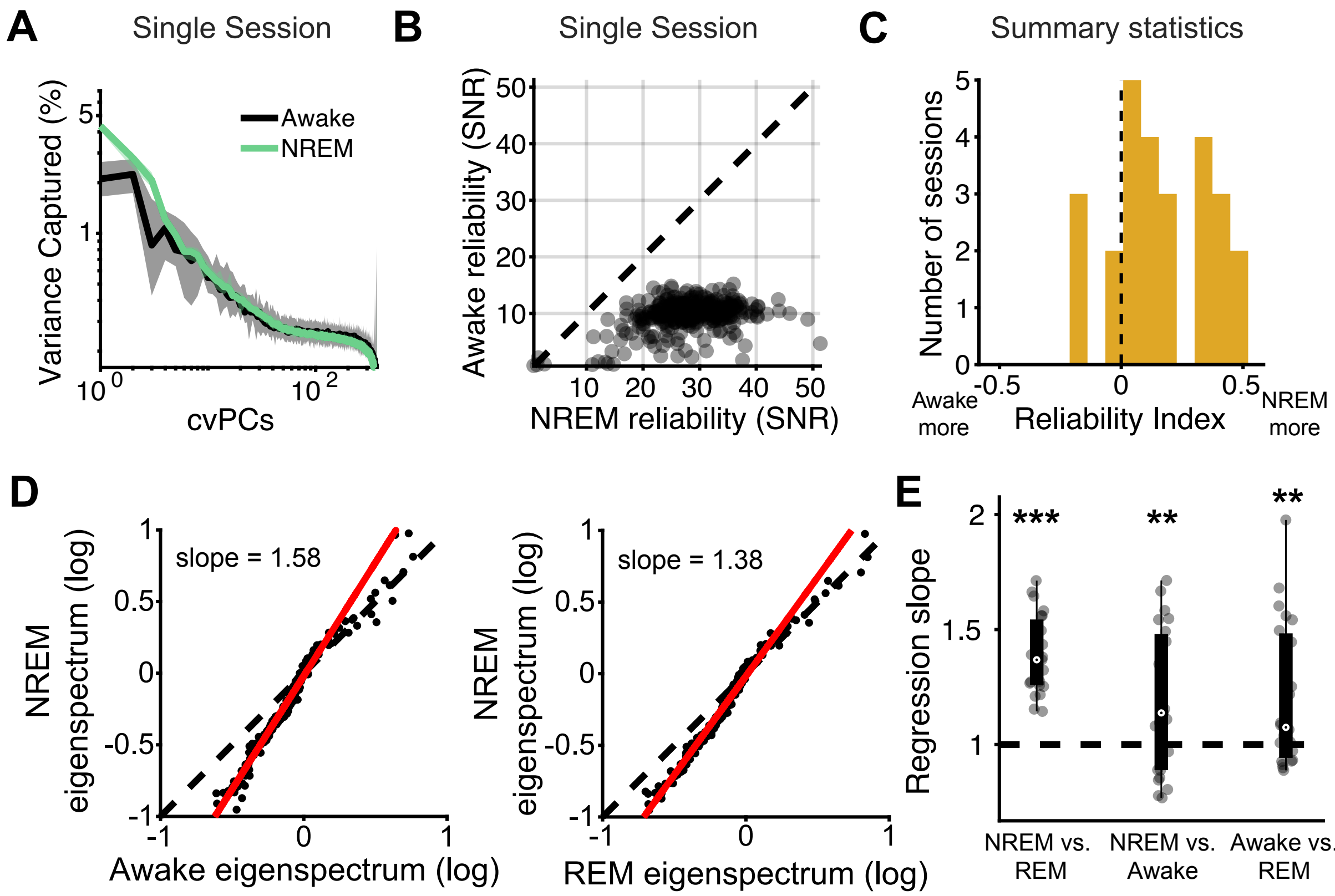

Fig. S 2. V1 population activity is more reliable and lower dimensional in NREM sleep than during wakefulness or REM sleep states. (**A**) Cross-validated principal components analysis (cvPCA) eigenspectra estimated from five adjacent segments of data show more segment-to-segment variability in wakefulness than in NREM (note wider confidence interval), suggesting NREM provides a more reliable estimate of population structure. (**B**) Single session example showing that individual eigenvalues from panel A are estimated more reliably from NREM than from awake spontaneous activity. (**C**) Across session summary showing that cvPCA eigenspectra can be estimated more reliably from NREM than wakefulness ($p=4.40 \times 10^{-4}$, $t(25)=4.05$). (**D**) An example session illustrating that the eigenspectrum decays faster (positive slope) in NREM, indicating lower-dimensional population activity. (**E**) Across multiple recording sessions in V1, NREM had the lowest dimensionality (NREM vs. REM: $p=2.15\times10^{-10}$; NREM vs Awake: $p=0.01$, Awake vs. REM: $p=0.005$, $n = 23$ recordings, paired t-test, two-sided, against the mean of 1, i.e. equal dimensionality). All box plots show median, 25th–75th percentiles, and 1.5× IQR whiskers.

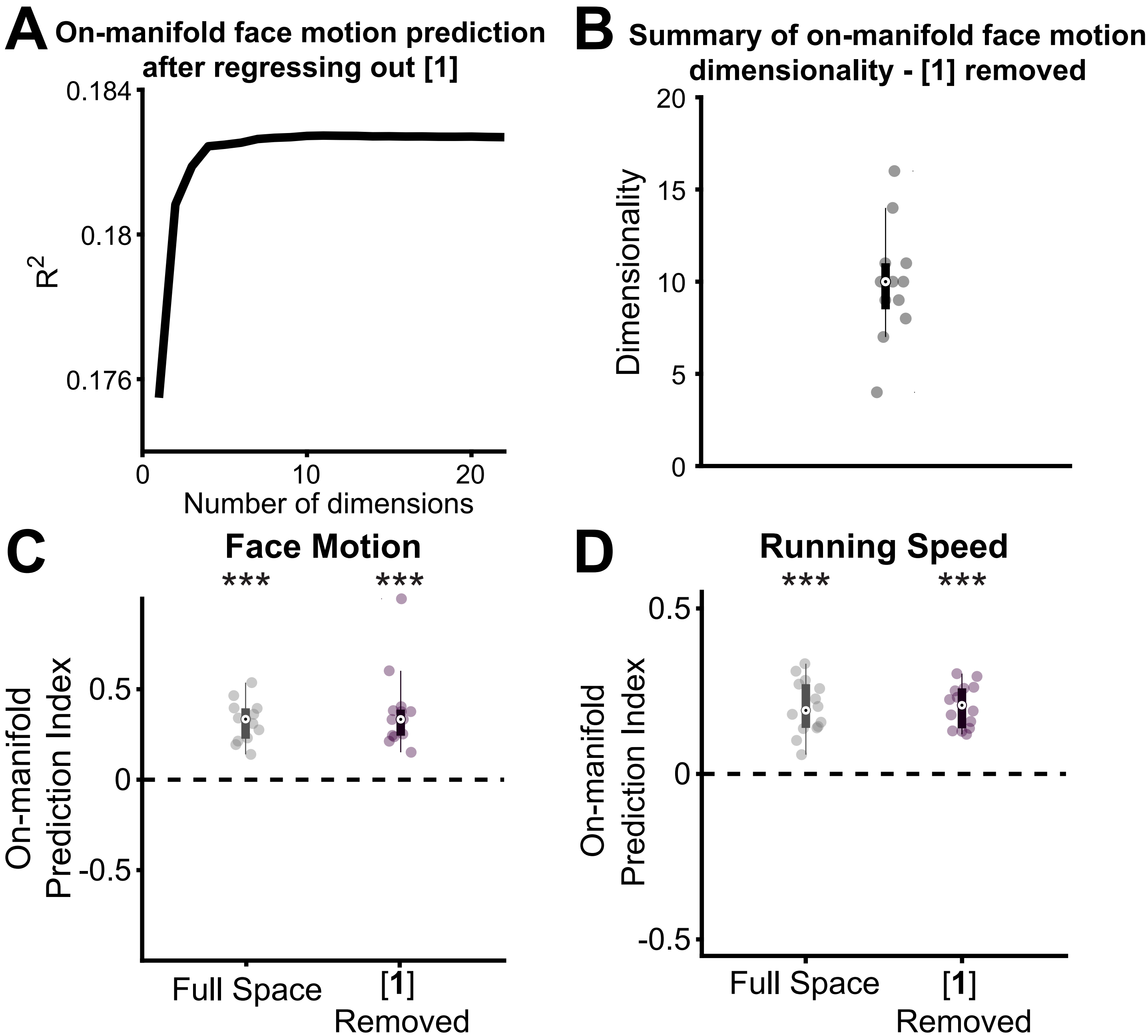

Fig. S 3. On-manifold prediction of movements is multidimensional and not mediated by global firing rate fluctuations. (**A**) Single session example illustrating that even after subtracting out global firing rate fluctuations, a multidimensional movement representation remains. (**B**) Across multiple sessions (n = 13), multidimensional movement representations remain after removing global firing rate fluctuations. [two-tailed t-test, ***p<0.001] (**C**) To test the possibility that on-manifold movement coding is mediated by both movements and spontaneous fluctuations affecting the global firing rate, we repeated our analyses after regressing out the [1] vector. This did not reduce on-manifold prediction performance. Prediction index, original: 0.32 ± 0.03, $p=2.45\times10^{-7}$ - [1] removed: 0.38 ± 0.06, $p=4.43\times10^{-5}$, mean ± s.e.m., n = 13 recordings sessions. One sample t-test, two-sided. (**D**) Same as **C**, but for running speed. Prediction index, original: 0.20 ± 0.02, $p=5.67\times10^{-7}$; [1] removed: 0.20 ± 0.02, $p=2.14\times10^{-8}$, mean ± s.e.m., n = 14 recording sessions. One sample t-test, two-sided. All box plots show median, 25th–75th percentiles, and 1.5× IQR whiskers.

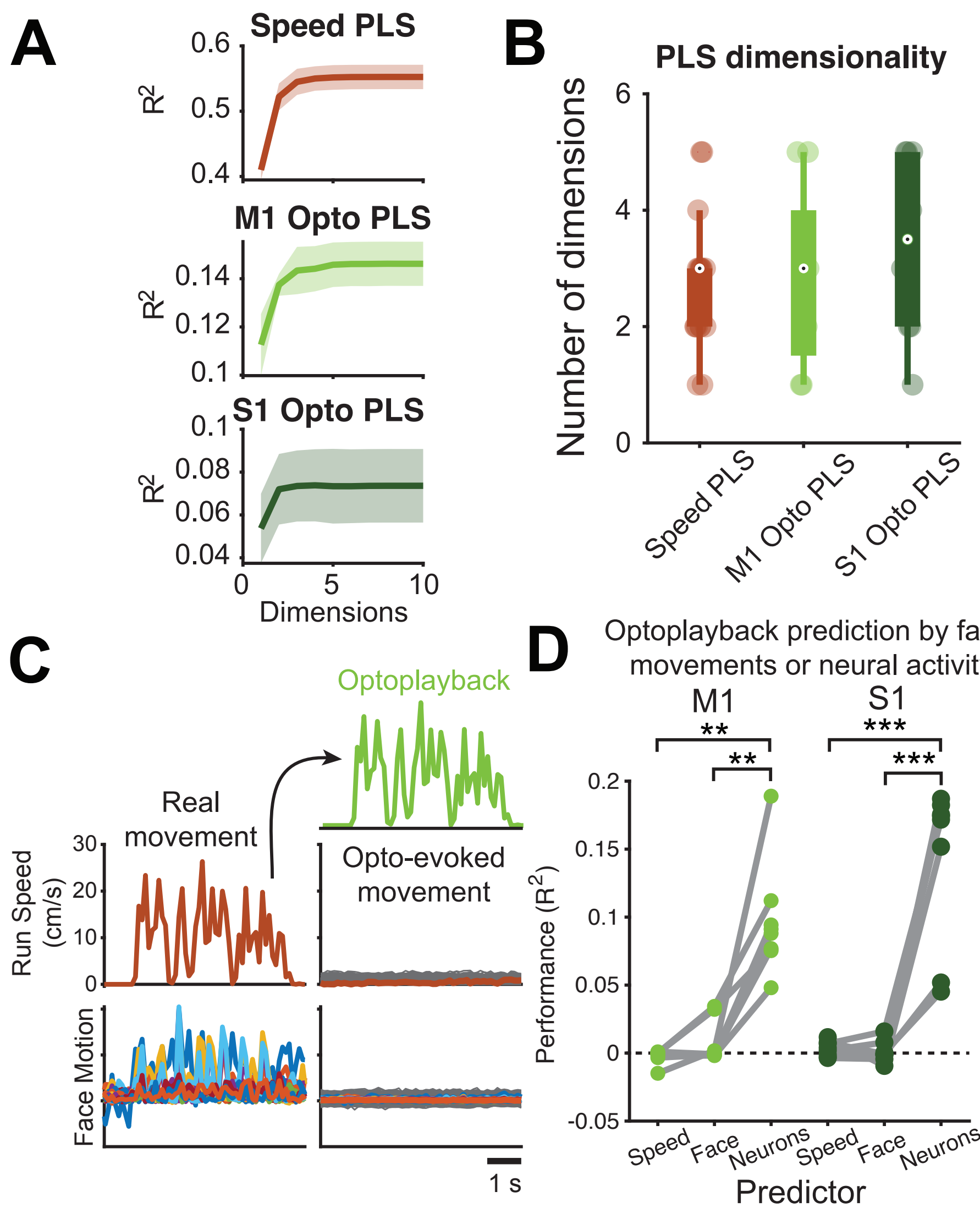

Fig. S 4. Optoplaybacks evoke multidimensional activity that is not explainable by opto-evoked movements. (**A**) Similar to running speed, M1 and S1 optoplaybacks created multidimensional representations in V1 neuronal activity. (**B**) A summary of PLS dimensionality shows similar dimensionality across running speed and M1 and S1 optoplayback of running speed. Dimensionality: running speed 2.70 ± 0.28, n = 17 recordings, M1 optoplayback 2.87 ± 0.55, n = 8 recordings 3.40 ± 0.45, n = 10 recordings. (**C**) (left) Example of real running speed and the first 16 facial motion PCs during a bout of spontaneous running. (right) Optoplayback of the running speed does not evoke running or face motion. Colored lines indicate optoplayback-triggered average movement, and gray lines indicate time-shuffled controls. (**D**) If optoplayback did generate movements that secondarily created neuronal representations, then we would be able to predict the optoplayback waveform from running speed or facial movements. On the contrary, neuronal activity predicts the optoplayback significantly better than movements. M1 optoplayback prediction: running speed -0.002 ± 0.002, face motion 0.008 ± 0.006, V1 neurons 0.10 ± 0.01, n = 8 recordings. S1 Optoplayback prediction: running speed 0.003 ± 0.002, face motion 0.001 ± 0.003, V1 neurons 0.14 ± 0.02, n = 8 recordings. M1 optoplayback prediction by face motion vs V1 neuronal activity $p = 1.80 \times 10^{-3}$, running speed vs V1 neuronal activity $p = 1.23 \times 10^{-3}$, S1 optoplayback prediction by face motion vs V1 neuronal activity $p = 5.99 \times 10^{-4}$, running speed vs V1 neuronal activity $p=7.04 \times 10^{-4}$. All tests are RM Anova, two sided, Tukey's post hoc. **p<0.01, ***p<0.001 . All box plots show median, 25th–75th percentiles, and 1.5× IQR whiskers

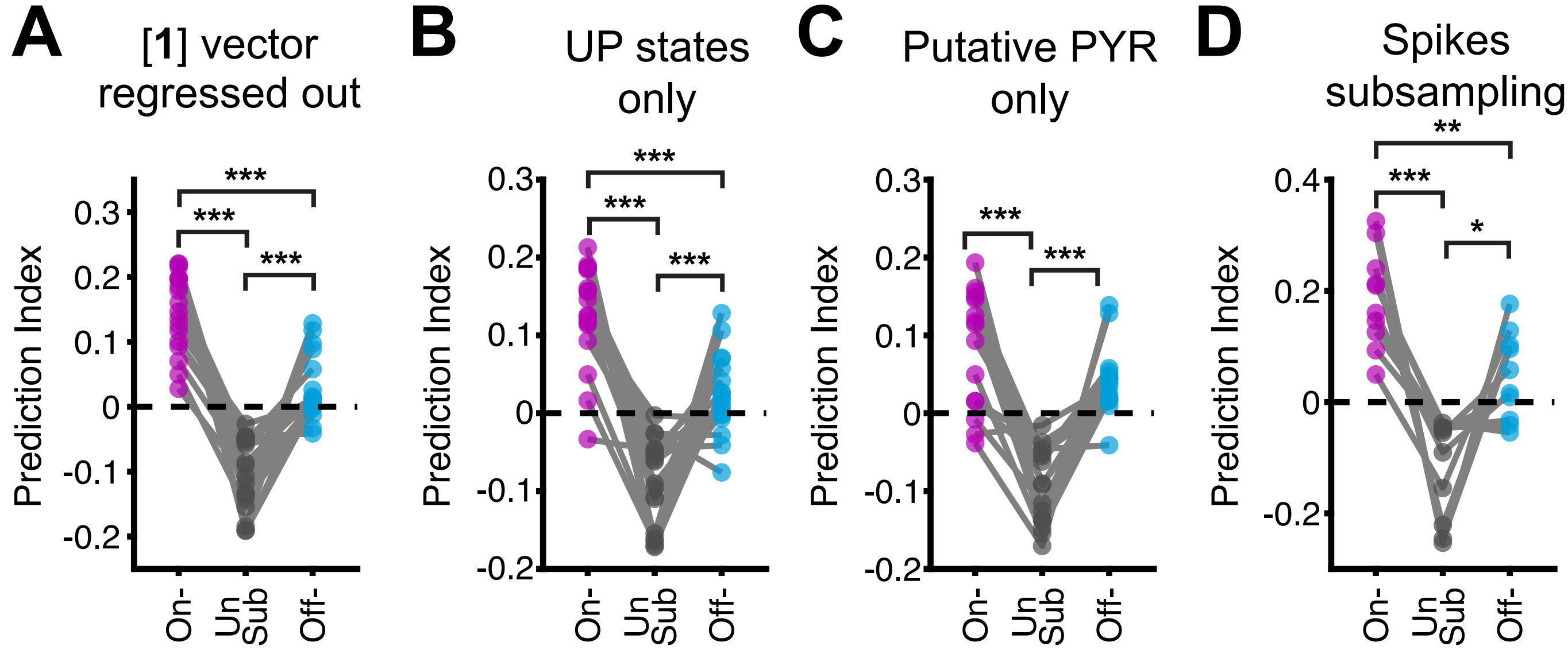

Fig. S 5. Off-manifold encoding of natural visual scenes is not explained by various nonspecific effects. Off-manifold coding remains intact after: (**A**) Regressing out global firing rate changes (the [**1**] vector) from the neuronal activity (Prediction Index: on-manifold 0.15 ± 0.01, unstructured -0.10 ± 0.01, off-manifold 0.03 ± 0.01, mean ± s.e.m., n=18 recordings. Repeated Measures ANOVA $F(2,34)=67.74$, $p=1.38 \times 10^{-12}$). (**B**) Estimating NREM cvPCs using UP state periods only (Prediction Index: on-manifold 0.13 ± 0.02, unstructured -0.09 ± 0.01, off-manifold 0.02 ± 0.01, mean ± s.e.m., n=18 recordings. Repeated Measures ANOVA $F(2,34)=46.38$, $p=1.92 \times 10^{-10}$). (**C**) Restricting the analysis to putative pyramidal cells. (Prediction Index: on-manifold 0.08 ± 0.02, unstructured -0.10 ± 0.01, off-manifold 0.04 ± 0.01, mean ± s.e.m., n=15 recordings. Repeated Measures ANOVA $F(2,28)=29.45$, $p=1.3 \times 10^{-7}$) (**D**) Subsampling the spikes in high firing rate neurons to match low firing rate neurons (Prediction Index: on-manifold 0.19 ± 0.03, unstructured -0.12 ± 0.03, off-manifold 0.05 ± 0.02, mean ± s.e.m., n=10 recordings. Repeated Measures ANOVA $F(2,18)=22.58$, $p=1.24 \times 10^{-5}$). [All tests are two sided, Tukey's post hoc *p<0.05, **p<0.01, ***p<0.001]. All box plots show median, 25th–75th percentiles, and 1.5× IQR whiskers.

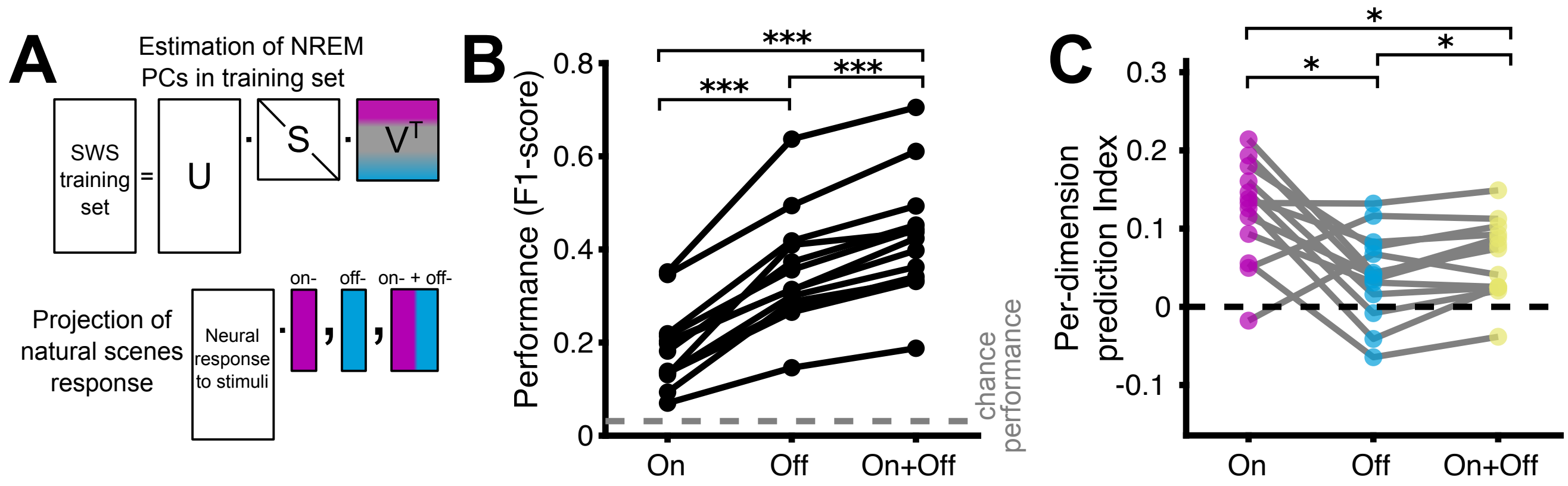

Fig. S 6. The on- and off-manifold subspaces encode complementary information about natural scene stimuli. (**A**) To test whether the on- and off-manifold subspaces encode redundant information, we tested the performance of a classifier in predicting stimulus identity from neuronal activity in the on-manifold subspace, the off-manifold subspace, or both combined. (**B**) Prediction performance is higher for both subspaces combined, suggesting they encode complementary stimulus information. (F1-score: on-manifold 0.19 ± 0.02, off-manifold 0.35 ± 0.03, on- and off-manifold 0.42 ± 0.04, mean ± s.e.m., n=13 recordings) Repeated Measures ANOVA $F(2,24)=148.99$, $p=2.94 \times 10^{-14}$ (**C**) If the on- and off-manifold subspaces encoded redundant information, then the per-dimension prediction index for the combined subspace would be decreased. This is because, unlike the analysis in panel B, the per-dimension prediction index is normalized for the number of dimensions. We observed an increase in per-dimension prediction index from off-manifold to combined on- and off-manifold, indicating that the on- and off-manifold subspaces are not encoding redundant information (Per-dimension prediction Index - on-manifold 0.12 ± 0.02, off-manifold 0.04 ± 0.02, on/off-manifold combined 0.06 ± 0.01, mean ± s.e.m., n=13 recordings. Repeated Measures ANOVA $F(2,24)=10.79$, $p=4.55 \times 10^{-4}$). [All tests are two sided, Tukey's post hoc *p<0.05, ***p<0.001]

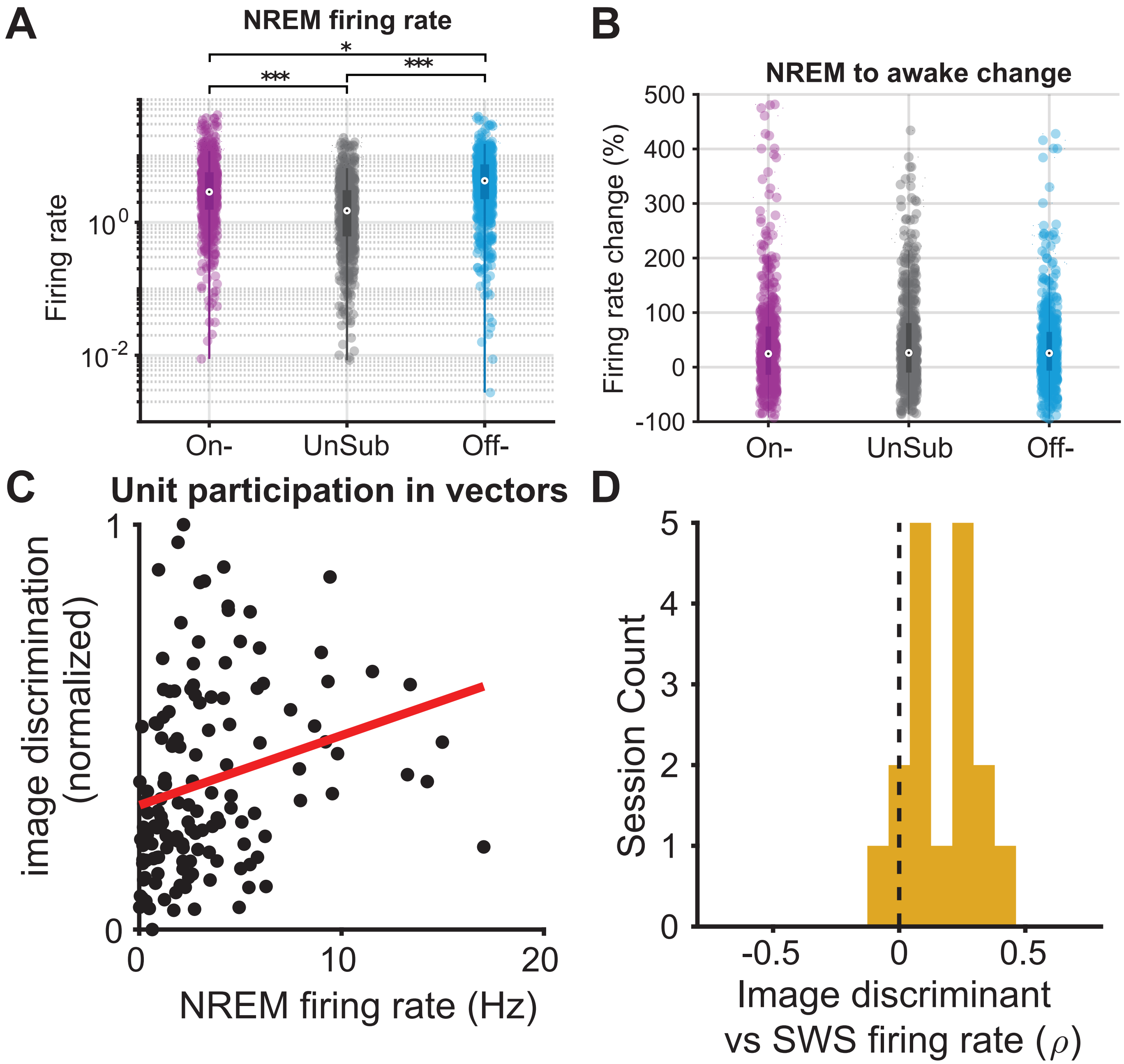

Fig. S 7. Off-manifold stimulus coding is not mediated by cells with low activity during NREM. (**A**) The top 20% of units with high magnitude loadings in on- or off-manifold participation have higher firing rates during NREM than units with strong unstructured subspace participation (average firing rate in spikes/s: on-manifold 4.8 ± 0.24, unstructured 2.4 ± 0.11, off-manifold 5.54 ± 0.21, mean ± s.e.m., n=594 neurons. One-way ANOVA $F(2,1779)=70.40$, $p=3.77 \times 10^{-30}$). (**B**) Units with strong on-, off-manifold, or unstructured subspace participation show similar changes in firing rate with wakefulness (all comparisons nonsignificant; sleep to awake % change - on-manifold: 62.93 ± 8.07, unstructured: 102.15 ± 33.78, off-manifold 56.97 ± 17.10, mean ± s.e.m., n=594 neurons). (**C**) An example session illustrating that units with stronger contributions to stimulus decoding exhibit higher firing rates during NREM (Pearson correlation=0.25, p=0.003; linear regression slope=0.017, p=0.003). (**D**) This correlation holds across multiple recording sessions in V1 (Pearson correlation 0.16 ± 0.03, mean ± s.e.m., n = 17 recording sessions, one sample t-test, two sided, $t(16)=4.91$, $p=1.56\times10^{-4}$). [All tests are two sided, Tukey's post hoc unless otherwise stated, *p<0.05, **p<0.01, ***p<0.001] All box plots show median, 25th–75th percentiles, and 1.5× IQR whiskers.

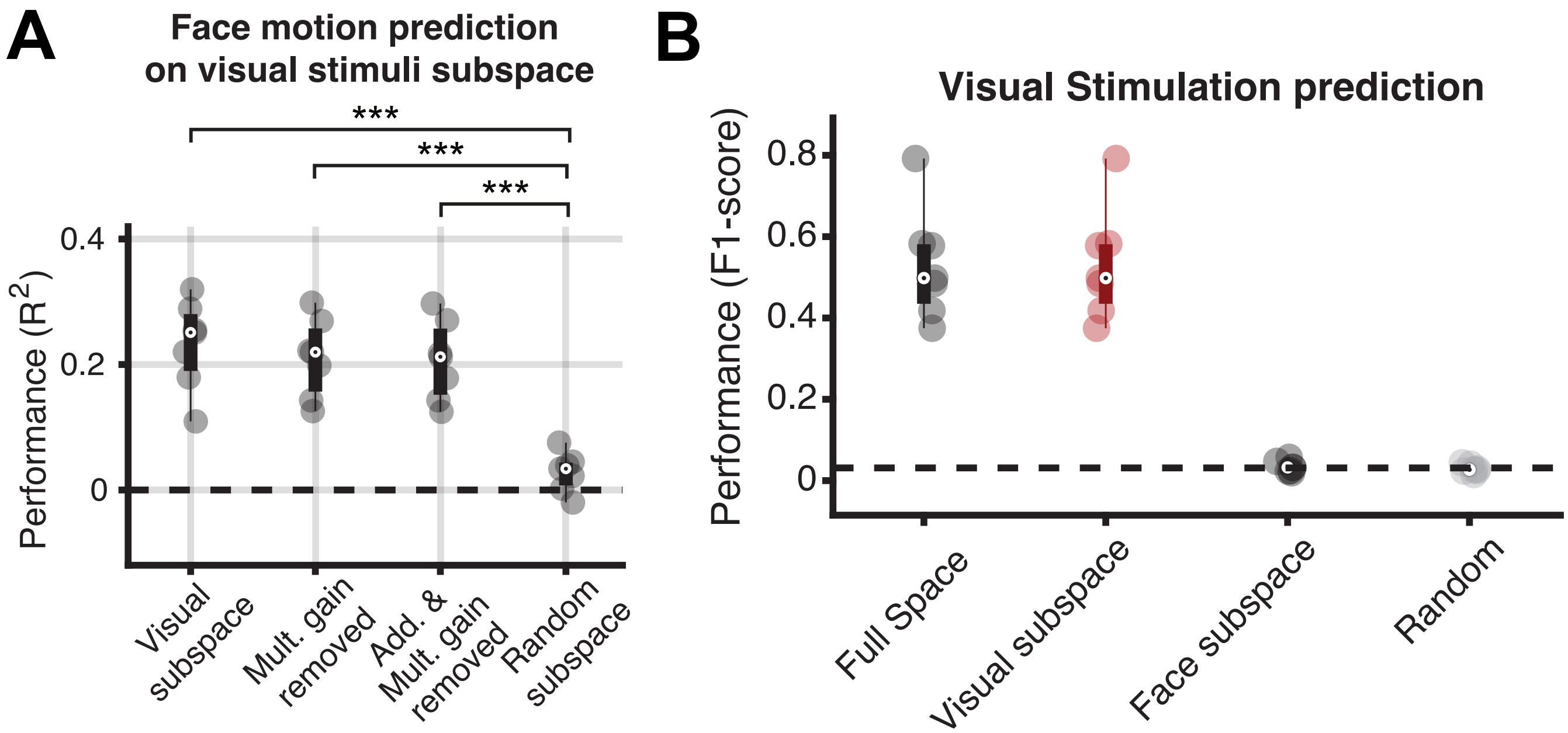

Fig. S 8. Face motion prediction in the stimulus encoding subspace is not due to multiplicative gain modulation. (**A**) Testing whether movement and stimulus representations interact requires each representation to be estimated without contamination by the other, or else there could be a false positive result. We record movement-evoked activity in the absence of visual stimuli, but some movements are inevitably present during visual stimulus presentations. We therefore estimate the stimulus-encoding subspace based solely on stimulus responses averaged over many trials, which should eliminate the effects of movement. However, we wished to confirm that the interaction we observe is not attributable to the well-established phenomenon of movement-evoked multiplicative gain modulation. We therefore fit single-neuron models of gain modulation based on face motion and regressed out these effects from each trial to obtain corrected estimates of the stimulus-encoding subspace. These corrected stimulus-encoding subspaces encoded facial motion to the same extent as the original estimate, which was substantially more than a random subspace (Multiplicative gain: Multi. Gain, Multiplicative Gain with additive term: Add. & Mult. gain). This suggests that multiplicative gain modulation is unlikely to account for the movement-stimulus interactions we observe (Fig. 6B). Repeated Measures ANOVA $F(3,18)=63.83$, $p=8.64\times10^{-10}$ (**B**) Unlike face motion prediction from the stimulus-encoding subspace, stimulus identity cannot be predicted from the face motion-encoding subspace. [All tests are two-sided, Tukey's post hoc *p<0.05, **p<0.01, ***p<0.001] All box plots show median, 25th–75th percentiles, and 1.5× IQR whiskers.

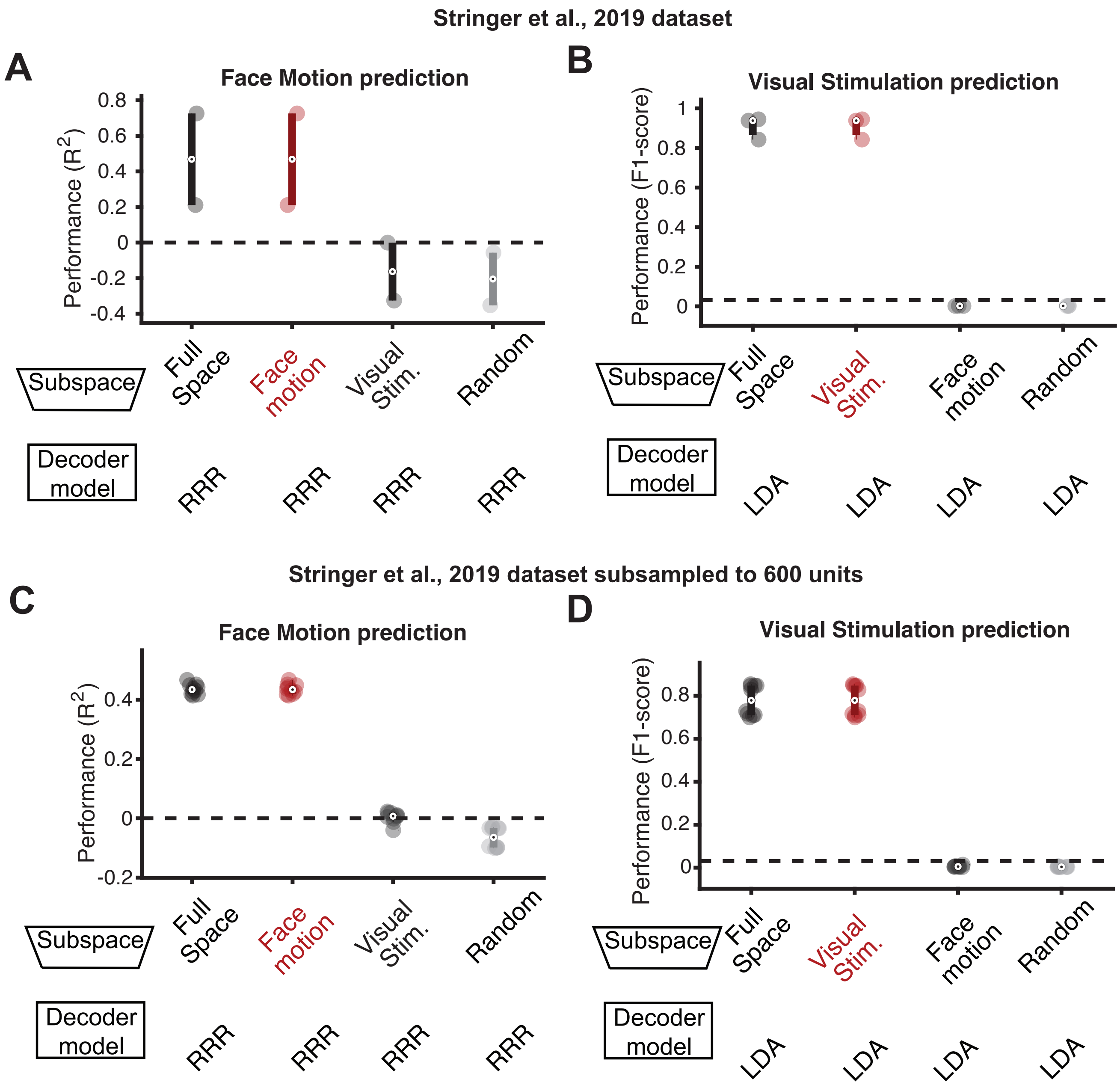

Fig. S 9. Additive visual stimulus and face motion interaction is not observed in the Stringer et al. 2019 dataset. We tested whether the face motion and stimulus interaction we observed (Fig. 6) could be replicated in a publicly available dataset with large-scale calcium imaging of superficial layers of mouse V1. (**A**) We applied the same analysis used on our data (Fig. 6B), but we did not observe successful face motion decoding in the Stringer dataset. The full space and face motion subspace are shown for comparison. We show only sessions that passed our criteria of performance above $R^2 > 0.2$. (**B**) Consistent with Stringer et al.'s published results and our analysis of our own data (Fig. S8B), we could not predict stimulus identity from the face motion subspace. Full space and visual stimulus subspace shown for comparison. (**C-D**) To test whether the different results in our dataset and Stringer et al.'s could be explained by the large number of neurons they imaged, we randomly subsampled the neurons (600 picked at random, repeated 5 times) and repeated the analyses. We used 600 neurons per session because this was the number required to obtain similar predictions of face motion to our dataset. The results with 600 neurons were identical to those from the full dataset. All box plots show median, 25th–75th percentiles, and 1.5× IQR whiskers.

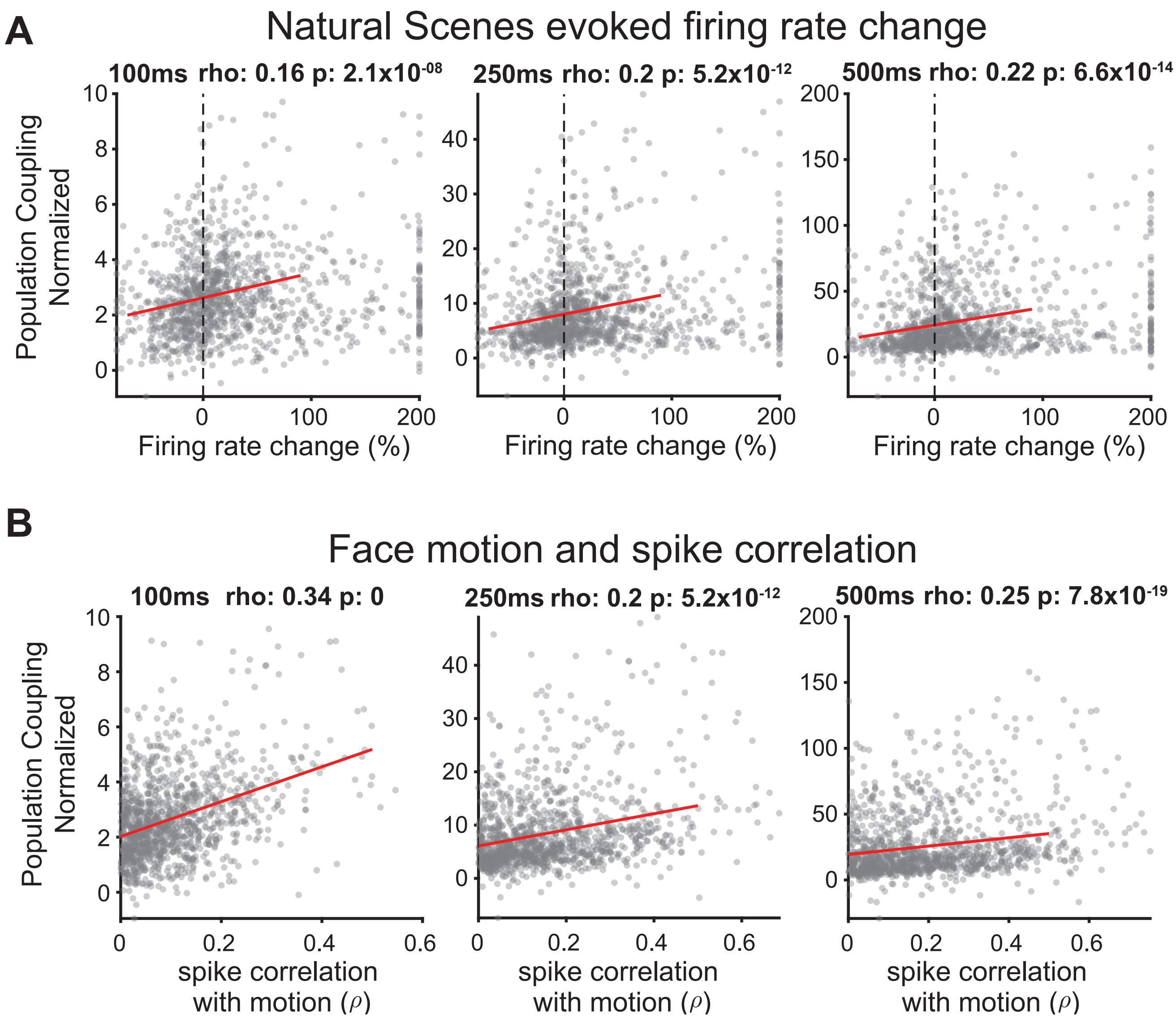

Fig. S 10. Chorister neurons in V1 exhibit stronger modulation by movement and visual stimuli at a range of timescales. (**A**) Units with stronger population coupling exhibit stronger modulation by visual stimuli when spike trains are binned with three different bin sizes. n=1180 neurons) (**B**) Units with stronger population coupling exhibit stronger modulation by facial movements when spike trains are binned with three different bin sizes. n =1187 neurons

Supplementary Table

Panel	Notes	test used	value	CI	n	df	P-value
1D	–	t-test	t=23.24	0.32 - 0.38	26	25	1.90×10^{-18}
2E	Face motion	RM ANOVA	F=62.64	-	13	(2,24)	2.98×10^{-10}
2E	Face motion on- vs UnSub	Multiple Comparisons	-	0.34 - 0.59	13	-	1.04×10^{-6}
2E	Face motion on- vs off-	Multiple Comparisons	-	0.31 - 0.60	13	-	8.18×10^{-6}
2E	Face motion UnSub vs off-	Multiple Comparisons	-	-0.12 - 0.09	13	-	9.3×10^{-1}
2H	Running speed	RM ANOVA	F=42.47	-	14	(2,26)	6.43×10^{-9}
2H	Running speed on- vs UnSub	Multiple Comparisons	-	0.18 - 0.33	14	-	2.59×10^{-6}
2H	Running speed on- vs off-	Multiple Comparisons	-	0.15 - 0.31	14	-	1.55×10^{-5}
2H	Running speed UnSub vs off-	Multiple Comparisons	-	-0.10 - 0.06	14	-	7.77×10^{-1}
3B	RNN FF1 input	RM ANOVA	F=12.78	-	10	(2,18)	3.5×10^{-4}
3B	on- vs UnSub	Multiple Comparison	-	0.01 - 0.28	10	-	3.7×10^{-2}
3B	on- vs off-	Multiple Comparison	-	0.13 - 0.28	10	-	8.75×10^{-5}
3B	UnSub vs off-	Multiple Comparison	-	-0.07 - 0.19	10	-	0.19
3B	RNN FF2 input	RM ANOVA	F=30.76	-	10	(2,18)	1.56×10^{-6}
3B	on- vs UnSub	Multiple Comparison	-	0.08 - 0.30	10	-	2.05×10^{-3}
3B	on- vs off-	Multiple Comparison	-	0.21 - 0.30	10	-	1.79×10^{-7}
3B	UnSub vs off-	Multiple Comparison	-	-0.05 - 0.18	10	-	0.31
3E	FF1 model: FF2 subspace vs. Random subspace	t-test bonferroni corrected	5.48	0.17 - 0.50	10	9	7.8×10^{-4}
3E	FF1 model - FF2 subspace vs. FF2 model - full space	t-test bonferroni corrected	9.83	0.41 - 0.73	10	9	8.26×10^{-6}
3H	M1 optoplayback	RM ANOVA	F=29.29	-	9	(2,16)	4.49×10^{-6}
3H	on- vs UnSub	Multiple Comparison	-	0.38 - 0.88	9	-	2.43×10^{-4}
3H	on- vs off-	Multiple Comparison	-	0.24 - 0.85	9	-	2.22×10^{-3}
3H	UnSub vs off-	Multiple Comparison	-	-0.28 - 0.12	9	-	0.52
3H	S1 optoplayback	RM ANOVA	F=17.71	-	9	(2,16)	8.79×10^{-5}
3H	on- vs UnSub	Multiple Comparison	-	0.21 - 0.95	9	-	5.40×10^{-3}
3H	on- vs off-	Multiple Comparison	-	0.33 - 0.69	9	-	1.12×10^{-4}
3H	UnSub vs off-	Multiple Comparison	-	-0.40 - 0.25	9	-	0.80
3J	running speed prediction cross subspace	Mixed model with random intercept	-	-	-	-	-
3J	Random subspace vs M1 subspace	Multiple Comparison	t=-5.14	-	6	25	3.34×10^{-4}
3J	M1 subspace vs S1 GPR	Multiple Comparison	t=2.87	-	6	25	0.078
3J	M1 GPR vs M1 subspace	Multiple Comparison	t=-1.73	-	6	25	0.52
3J	random subspace vs S1 subspace	Multiple Comparison	t=-7.31	-	6	25	1.62×10^{-6}
3J	M1 GPR vs S1 subspace	Multiple Comparison	t=-3.96	-	6	25	6.51×10^{-3}
3J	S1 GPR vs S1 subspace	Multiple Comparison	t=-5.09	-	6	25	3.8×10^{-4}
3K	face motion prediction cross subspace	Mixed model with random intercept	-	-	-	-	-
3K	Random subspace vs M1 subspace	Multiple Comparison	t=-7.80	-	8	22	5.09×10^{-7}
3K	Random subspace vs S1 subspace	Multiple Comparison	t=-9.68	-	9	22	1.24×10^{-8}
3L	M1 and S1 combined	t-test for mean of 1/32	t=-3.09	0.003 - 0.028	7	6	0.02
4D	Prediction index	RM ANOVA	F=31.23	-	13	(2,24)	2.09×10^{-7}
4D	on- vs UnSub	Multiple Comparisons	-	0.14 - 0.30	13	-	2.38×10^{-5}
4D	on- vs off-	Multiple Comparisons	-	0.017 - 0.15	13	-	0.013
4D	UnSub vs off-	Multiple Comparisons	-	-0.22 - -0.06	13	-	1.70×10^{-3}
5D	Population sparseness	RM ANOVA	F=198.56	-	11	(2,20)	6.42×10^{-14}
5D	on- vs UnSub	Multiple Comparisons	-	-0.060 - -0.026	11	-	1.31×10^{-4}
5D	on- vs off-	Multiple Comparisons	-	-0.17 - -0.13	11	-	1.01×10^{-8}
5D	UnSub vs off-	Multiple Comparisons	-	-0.13 - -0.08	11	-	1.22×10^{-6}

table S 1. Summary of statistics for the main figures

5F	Population coupling vs Subspace participation	RM ANOVA	F=264.51	-	13	(2,24)	4.46×10^{-17}
5F	on- vs UnSub	Multiple Comparisons	-	0.64 - 0.91	13	-	9.42×10^{-9}
5F	on- vs off-	Multiple Comparisons	-	-0.19 - -0.06	13	-	8.90×10^{-4}
5F	UnSub vs off-	Multiple Comparisons	-	-1.03 - -0.77	13	-	3.39×10^{-9}
5G	Pairwise correlation	RM ANOVA	F=169.68	-	17	(2,32)	9.24×10^{-18}
5G	on- vs UnSub	Multiple Comparisons	-	0.09 - 0.14	17	-	2.63×10^{-8}
5G	on- vs off-	Multiple Comparisons	-	0.12 - 0.15	17	-	1.18×10^{-9}
5G	UnSub vs off-	Multiple Comparisons	-	8.20×10^{-4} - 0.03	17	-	0.04
5G	Soloist - chorister index	RM ANOVA	F=248.05	-	17	(2,32)	3.30×10^{-20}
5G	on- vs UnSub	Multiple Comparisons	-	0.27 - 0.37	17	-	1.25×10^{-9}
5G	on- vs off-	Multiple Comparisons	-	-0.046 - 0.008	17	-	0.21
5G	UnSub vs off-	Multiple Comparisons	-	-0.39 - -0.29	17	-	1.19×10^{-9}
6B	Face motion prediction	RM ANOVA	F=30.05	-	7	(4,24)	4.96×10^{-9}
6B	Visual stim. vs Random	Multiple Comparisons	-	0.12 - 0.28	7	-	4.67×10^{-4}
6B	Visual stim. vs Visual stim. on-	Multiple Comparisons	-	-0.026 - 0.14	7	-	0.19
6B	Visual stim. vs Visual stim. UnSub	Multiple Comparisons	-	0.10 - 0.28	7	-	1.19×10^{-3}
6B	Visual stim. vs Visual stim. off-	Multiple Comparisons	-	0.13 - 0.25	7	-	1.65×10^{-4}
6B	Random vs Visual stim. on-	Multiple Comparisons	-	-0.27 - -0.03	7	-	0.018
6B	Random vs Visual stim. UnSub	Multiple Comparisons	-	-0.059 - 0.039	7	-	0.94
6B	Random vs Visual stim. off-	Multiple Comparisons	-	-0.061 - 0.031	7	-	0.73
6B	Visual stim. on- vs Visual stim. UnSub	Multiple Comparisons	-	-0.007 - 0.28	7	-	0.062
6B	Visual stim. on- vs Visual stim. off-	Multiple Comparisons	-	0.018 - 0.25	7	-	0.027
6B	Visual stim. UnSub vs Visual stim. off-	Multiple Comparisons	-	-0.074 - 0.06	7	-	0.99
6C	Motion modulation vs. population coupling	Spearman correlation	ρ=0.34	-	1187	1185	$< 1.0 \times 10^{-18}$
6C	Visual stimuli vs. population coupling	Spearman correlation	ρ=0.25	-	1180	1178	7.64×10^{-18}
6D	Image discrimination vs. face motion	t-test	t=5.40	0.12 - 0.30	10	9	4.32×10^{-4}
6E	Face motion prediction	RM ANOVA	F=20.48	-	7	(2,12)	1.35×10^{-4}
6E	Full space vs soloist	Multiple Comparisons	-	-0.006 - 0.079	7	-	0.09
6E	Full space vs chorister	Multiple Comparisons	-	0.042 - 0.11	7	-	1.16×10^{-3}
6E	Soloist vs chorister	Multiple Comparisons	-	0.008 - 0.074	7	-	0.02

table S 1. Summary of statistics for the main figures
